# Self-Assembly of Unconventional Triphenylene-Based Frustrated Amphiphile in Solution

**DOI:** 10.1021/acs.langmuir.5c05203

**Published:** 2026-02-06

**Authors:** Henrique Musseli Cezar, Giacomo Berton, Tommaso Lorenzetto, Sandro Zorzi, Cedrix J. Dongmo Foumthuim, Szymon Mikołaj Szostak, Pablo Ballester, Claudia Mondelli, Ralf Schweins, Viviana Cristiglio, Fabrizio Fabris, Reidar Lund, Alessandro Scarso, Achille Giacometti, Michele Cascella

**Affiliations:** † Hylleraas Centre for Quantum Molecular Sciences, 6305 University of Oslo, PO Box 1033 Blindern, 0315 Oslo, Norway; ‡ Department of Chemistry, 6305 University of Oslo, PO Box 1033 Blindern, 0315 Oslo, Norway; ¶ Dipartimento di Scienze Molecolari e Nanosistemi, Università Ca’ Foscari Venezia, via Torino 155, 30172 Venezia, Italy; § Fondazione Bruno Kessler, Centre for Sustainable Energy, Via Sommarive 18, 38123 Povo (TN), Italy; ∥ Department of Chemical Sciences, University of Padova, via Marzolo 1, 35131 Padova, Italy; ⊥ Institute of Chemical Research of Catalonia (ICIQ), Barcelona Institute of Science and Technology, Avinguda Països Catalans 16, Tarragona 43007, Spain; # Catalan Institution for Research and Advanced Studies (ICREA), Passeig Lluis Companys 23, Barcelona 08010, Spain; ○ CNR-IOM, Institut Laue Langevin, 71 Avenue des Martyrs, 38042 Grenoble Cedex 9, France; ◆ Institut Laue Langevin, 71 Avenue des Martyrs, 38042 Grenoble Cedex 9, France; ∇ European Centre for Living Technology (ECLT) Ca’ Bottacin, Dorsoduro 3911, 30123 Venice, Italy; ▼ Donostia International Physics Centre (DIPC), Manuel Lardizabal Ibilbidea, 4, 20018 Donostia-San-Sebastián, Gipuzkoa, Spain

## Abstract

Unconventional amphiphilic molecules having a rigid hydrophobic central unit and flexible or semiflexible hydrophilic chains on the rim have proven valuable in several technological applications, for example, host–guest chemistry. The further presence of alternating hydrophobic and hydrophilic tails complicates the definition of clear core and shell regions, resulting in complex segregation and frustrated self-assembly. In this study, we investigate symmetric triphenylene-based amphiphilic derivatives with alternating benzyl and alkylsulfonate groups. We characterize their self-assembly in water and different solutions using several experimental techniques, including NMR, atomic force microscopy, dynamic light scattering, and small-angle X-ray and neutron scattering, alongside extensive molecular dynamics simulations, including both atomistic and coarse-grained integrative modeling with metainference. In aqueous solutions, these amphiphiles form stacked assemblies, adopting alternate up–down conformations driven by π-stacking of up to six molecules. The introduction of NaCl salt screens unfavorable electrostatic interactions, thus promoting further π-stacking and leading to the formation of larger elongated aggregates.

## Graphical abstract

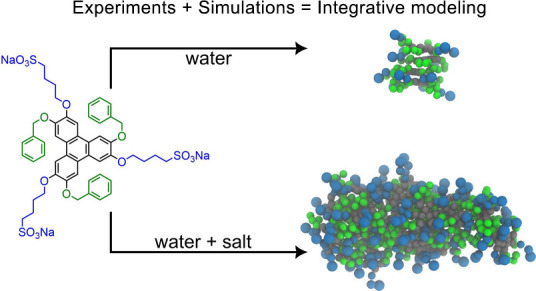

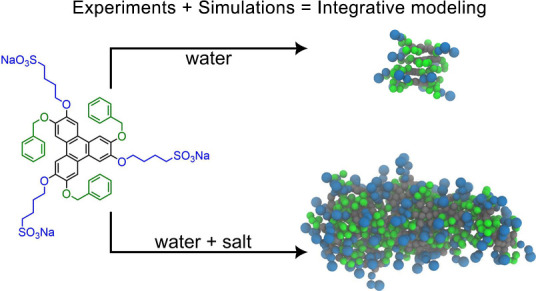

## 1 Introduction

Amphiphilic molecules in solution often self-assemble into supramolecular structures with various possible macromolecular organizations.^1–3^ The size and shape of these multimolecular objects are critical for their function and applications, for example, as drug carriers,^4^ templating nanoparticle synthesis,^5^ catalysis,^6^ or effective agents for cleaning works of art.^7^

Conventional surfactants typically consist of a rigid, globular-shaped hydrophilic head and flexible hydrophobic tails, as illustrated in Figure 1a. Depending on their geometric attributes,^8^ these surfactants often organize into spherical micelles, cylinders, fibers, or vesicles, and their self-assembly is commonly rationalized on the basis of the packing parameter.^9^ However, real systems are much more complex, and dedicated experiments and detailed numerical simulations are usually necessary to understand the detailed properties and driving forces responsible for aggregation.^10,11^ For instance, some of us have recently shown that even relatively simple well-known surfactants such as Triton X-100 display a complex zoo of assemblies deviating from those of conventional packing model predictions.^11^

**Figure 1:**
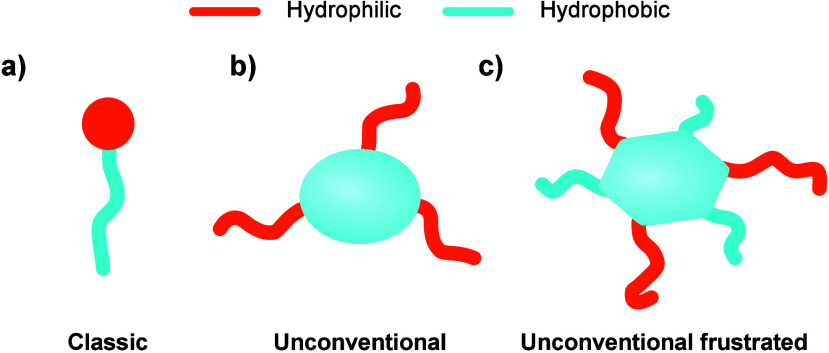
Schematic representation of some types of amphiphilic molecules. (a) Conventional: rigid hydrophilic headgroup and flexible hydrophobic chain; (b) unconventional: rigid hydrophobic unit and rigid hydrophilic chains; and (c) unconventional frustrated: rigid hydrophobic flat unit with alternating hydrophilic and hydrophobic units on the rim. The hydrophilic (orange) and hydrophobic (blue) moieties are colored differently.

Following the development of artificial self-assembling biomimetic systems in water, unconventional amphiphiles are emerging in the recent literature as useful tools for the exploration of the chemical and sequence space beyond that used routinely in biology.^12–18^ For some types of unconventional surfactants, such as those with the symmetric design illustrated in Figure 1b, the self-assembly process is relatively well understood, generally leading to capsules or nanocages,^19^ used to transport chemicals in supramolecular applications.^20–24^ A key feature of these amphiphilic molecules is the maintenance of some degree of separation between hydrophobic and hydrophilic regions.

The presence of alternating hydrophobic and hydrophilic segments leads to a balance between their chemical moieties, which can lead to interesting self-assembly and interfacial behaviors.^25^ Within the framework of surfactants, these are generally termed *frustrated surfactants*. The present study deals with a combination with the two above features, frustration and reverse polarity. In this case, henceforth termed *unconventional frustrated surfactants*, the presence of a central hydrophobic unit is coupled with flexible tails with alternating hydrophobic/hydrophilic characters. Much less is known in this case, despite their interesting potential applications. For example, lipopeptides possess a head that is not purely hydrophilic and exhibit different properties and assemblies even for small variations in composition, such as the addition or change of one or a few amino acids.^26,27^

Another class of even more complex frustrated amphiphilic molecules is characterized by a rigid flat core adorned with alternating hydrophobic and hydrophilic substituents, as illustrated in Figure 1c. For this specific case, only a few examples have been studied in the literature. Shionoya and collaborators reported the aggregation of six *C*_3_-symmetric hexa-substituted aromatic subcomponents with alternating aromatic pyridinium units assembling into nanometric cubic^28,29^ or tetrahedral^30^ structures, as a function of the presence of suitable guests. Different examples of amphiphilic units also with *C*_2_ symmetry led to similar cubic assemblies in polar protic solvents.^31–33^ A further example was provided by Yoshizawa and Catti^19^ based on a rigid tri-branched scaffold with an all-*syn* 1,3,5-trimethyl-2,4,6-tri­phenyl­benzene core forming a tetrameric capsule in water. For all of the cases described above, the determination of the assemblies formed in water is far from trivial.

The aim of this study is to shed new light on these self-assembly properties. To this end, we designed a symmetric amphiphilic benzylsulfonate (BZS) molecule with a rigid triphenylene^34,35^ hydrophobic core adorned with flexible alternating hydrophilic and hydrophobic substituents (see Figure 2). Triphenylene is known to form stacked aggregates as a result of its planar and aromatic nature. However, the effect of alternating substituents on these properties and aggregation is unclear. Although π-stacking could drive molecule assembly similar to chromonic liquid crystals,^36^ the presence of sulfonate groups indicates an electrostatic component affecting self-assembly, particularly in avoiding Coulomb repulsive interactions.

**Figure 2:**
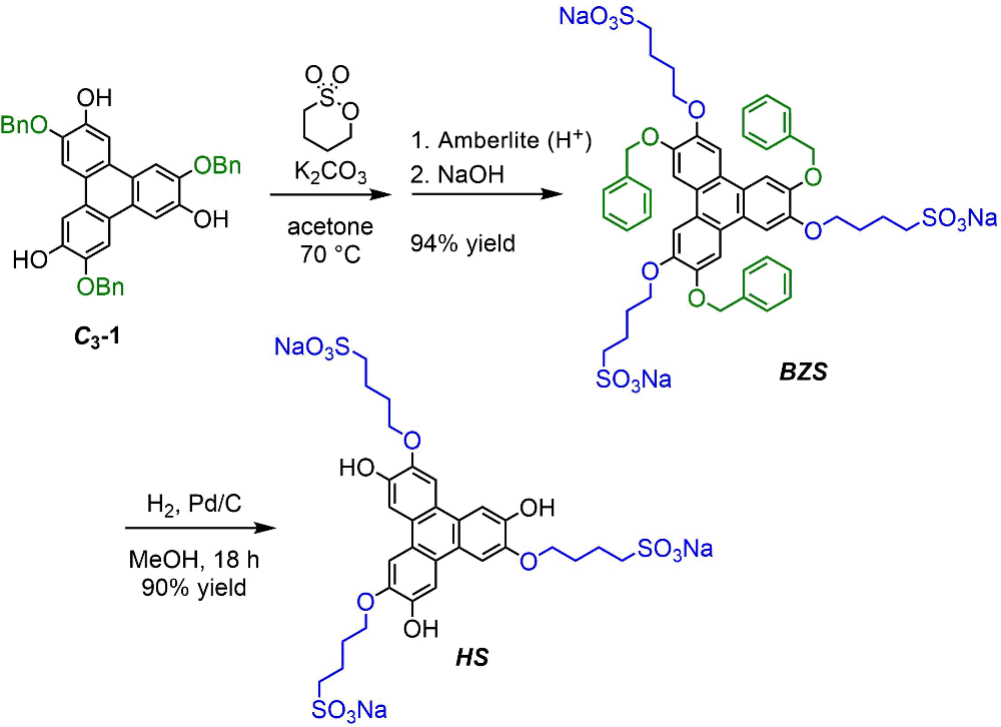
Synthesis of the negatively charged derivative BZS based on alternating benzyl and butylsulfonate units and the control compound HS.

To unravel the self-assembly of BZS, we combined ^1^H NMR, diffusion-ordered spectroscopy (DOSY) NMR, small-angle neutron scattering (SANS), and small-angle X-ray scattering (SAXS) experiments. In particular, SAXS experiments are used to investigate the role of electrostatic screening through salt addition in the aggregation process. The small-angle scattering experiments are interpreted using integrative modeling,^37,38^ combining experimental data and simulations, following our previous work.^11^ We use coarse-grained metainference^39^ simulations and atomistic molecular dynamics (MD) simulations, and we make no assumptions on the shape of the aggregates. The combination of experimental measurements and molecular modeling revealed strong environmental control on the self-assembled features, which vary from a small number of stacking units in pure water to larger, less organized formations upon the addition of salt.

## 2 Methods

### 2.1 Synthesis

The synthesis of BZS was obtained starting from the molecule labeled as *C*_3_-1 ^40^ through a one-step reaction with 1,4-butane sultone under basic conditions and subsequent ion exchange (Figure 2). By hydrogenation of BZS, an analogous *C*_3_-symmetric amphiphilic species, another molecule denoted as HS, was obtained. This molecule, lacking the benzyl hydrophobic unit, was used as a control reference, unable to assume the alternated orientation of the substituents in water (see Supporting Information, SI).

### 2.2 Characterization

#### 2.2.1 ^1^H NMR and DOSY

^1^H NMR, ^13^C NMR, and 2D spectra were recorded with Bruker Avance II 400, Ascend 400, and Ascend 500 spectrometers, operating at 400–500 and 100–126 MHz, respectively. Resonance frequencies are referred to tetramethylsilane. DOSY experiments were performed with the BPLED sequence, calibrating the diffusion time (Δ) in order to obtain a signal ratio of 20 between gpz = 2 and gpz = 95, and elaborated with Bruker Topspin and Dynamics Center.

#### 2.2.2 AFM and DLS

Atomic force microscopy (AFM) measurements were carried out on an Asylum Research Cypher S instrument on dry samples on mica. Dynamic light scattering (DLS) was performed on a Wyatt Technology DynaPro NanoStar instrument.

#### 2.2.3 Small-Angle Scattering

SAXS experiments were conducted at the BM29 beamline^41^ and ID02 beamline^42^ of the European Synchrotron Radiation Facility in Grenoble, France. Data acquisition at BM29 was performed using the Pilatus3 X 2M detector, operating at an energy of 12.5 keV in a vacuum environment. The employed photon wavelength was λ = 1 Å, resulting in a wave vector range of *q* = 0.005–0.5 Å^–1^, where *q* is defined as *q* = $\frac{4\pi \sin\left(\theta /2\right)}{\lambda }$ and θ represents the scattering angle. Samples with a volume of 50 μL were measured utilizing an autosampler, and the exposure was divided into ten 1 s frames; the corresponding buffer was measured both before and after each sample. Data acquisition at ID02 was performed using the Eiger2-4M detector with an operating energy of 18 keV, detector distance of 0.8 m, and photon wavelength λ = 1 Å, resulting in an accessible *q* range of *q* = 0.0178–1.78 Å^–1^. Buffers were measured before and after the sample. The intensity of the water scattering was employed to scale the data to absolute intensity. Postmeasurement, the frames were evaluated and averaged if no systematic deviations, such as those resulting from radiation damage, bubbles, or empty capillaries, were detected. Data reduction was performed in accordance with the instrument’s standard protocol.

All the SANS results reported in the following are based on data from an Institut Laue Langevin (ILL) beamtime proposal.^43^ The measure was obtained on the instrument D11 at a fixed wavelength of 0.6 nm, for three sample to detector distances (SDDs) of 1.2, 8, and 39 m, in order to cover a large region of scattering vector *q*, from 0.0025 to 0.5 Å^–1^. The transmission of the sample was obtained at an SDD of 8 m. The data reduction was performed with the software package LAMP.^44^ The calibration procedure was done by means of a H_2_O measurement to determine the absolute scale and the detector efficiencies.^45^ SANS measurements were also performed on the D16 diffractometer at the ILL as part of the experimental campaign. While these data are not shown in the present work, they are consistent with the D11 results reported here and are available through the ILL data repository.^43^

### 2.3 Modeling and Simulations

#### 2.3.1 Atomistic Molecular Dynamics

Atomistic MD simulations were performed with the GROMACS molecular package (v5.1.4, v2018.3, and v2018.7).^46^ Following the experimental conditions, 41 BZS (see Figure 3a) and 88 605 water molecules were randomly inserted into a cubic box of unit cell 14 nm to achieve a molecular concentration of 25 mM. We also added 121 Na^+^ counterions to the simulation box, which makes the system electroneutral. Atomic interactions were described using the all-atom version of the Optimized Potentials for Liquid Systems (OPLS-AA) force field.^47^ The 4-site rigid TIP4P water model^48^ was used to solvate the system. We anticipate that the largest stable cluster found within the simulation time scale of 50 ns was four, i.e., 4mer-BZS aggregate. To address the stability of the tetramer aggregates, we tested alternative preferred binding/interaction modes of the BZS. Hence, the coordinates of the tetrameric cluster obtained were extracted and used to build a 3 × 3 lattice of 4mer-BZS moieties randomly surrounded by adamantane (ADL) units (see SI). Altogether, the system includes 27 4mer-BZS units and 41 ADL solvated in a 12 nm cubic box, resulting in a molecular concentration of approximately 146 mM.

**Figure 3:**
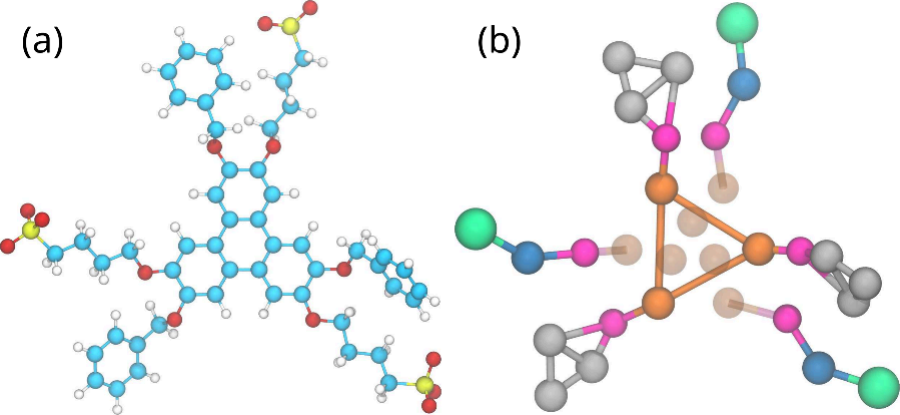
(a) BZS atomic structure and (b) respective coarse-grain mapping. For the atomic structure in (a), the color (atom) mapping is cyan (carbon), white (hydrogen), red (oxygen), and yellow (sulfur). The color (Martini 3 bead type) mapping in (b) is orange (TC5e), magenta (TN2a), blue (SC1), gray (TC5), and green (SQ4n). The semitransparent orange beads in (b) represent virtual sites whose positions are determined relative to the solid orange beads and, hence, not with “spring” bonds like the other bonds shown.

Minimization of the solvent was performed by keeping the positions of the BZS and ADL frozen. Subsequently, for each system considered herein, two consecutive rounds of temperature and pressure equilibrations in canonical *NVT* and isobaric–isothermal *NPT* ensembles were performed. In the first round of equilibration, the temperature was set to the reference value of 298.15 K using the *v*-rescale thermostat, with a coupling constant of 0.1 ps. The leapfrog integrator with an integration time step of 1 fs was used. In addition, the particle mesh Ewald summation was used to account for long-range electrostatics, with a real space cutoff of 12 Å. All solute heavy atoms were position restrained with a harmonic force constant of 1000 kJ mol^–1^ nm^–1^, and hydrogen bonds length were constrained with the LINCS algorithm.^49^ In the second equilibration round, the Parrinello–Rahman barostat^50^ was used to couple the system to the external pressure bath around 1.01325 bar with a coupling constant of 1 ps and the isothermal compressibility equal to 4.5 × 10^–5^ bar^–1^. Finally, in the production runs, the position restraints were released, and the systems were allowed to sample the entire phase space using an integration time step of 1 fs. A time scale of 50 ns was achieved for all atomistic simulations discussed herein, except for the one embedding ADL units, whose μs simulation time scale was reached.

#### 2.3.2 Coarse-Grained Molecular Dynamics

We use Martini 3 ^51^ to create a model for BZS following the guidelines for small molecule mapping.^52^ The choice of Martini 3 is based on the improved packing description of the model in comparison with previous versions,^51^ due to the possibility of using tiny (T) and small (S) beads, on top of the regular (R) bead types. The chosen mapping for BZS uses T and S beads and virtual sites for the central aromatic core, as shown in Figure 3b. The positions of the virtual sites are determined relative to the three TC5e (orange) sites that form the triangle represented by solid orange beads. The virtual sites keep the core planar without having to introduce extra constraints. We tried using TC5 beads instead of TC5e for the core beads, obtaining stronger stacking. Since the experiments showed a small number of molecules per aggregate, to avoid biasing for long stacks, we used TC5e for production runs. The water and ion beads followed the usual Martini 3 representation.

The coarse-grained MD simulations were performed using GROMACS 2019.5.^46^ The equations of motion were integrated with the leapfrog algorithm with a 20 fs time step. The simulations were carried out in the *NPT* ensemble with *T* = 298.15 K and *P* = 1.0 bar using the velocity-rescale thermostat with τ_*T*_ = 1.0 ps and the Bussi barostat with τ_*P*_ = 2.0 ps. As the SO_3_^–^ groups are charged, Na^+^ counterions were used to keep the box with a neutral charge, and a reaction field with ϵ = 62 was used to account for long-range electrostatics. A cutoff of 1.1 nm was used for both the Lennard-Jones and Coulomb interactions. Bonds related to the representations of the benzene moieties and the triangle of the central aromatic core shown in Figure 3 were constrained using the LINCS algorithm.^49^ We used a cubic simulation box with about 20.3 nm sides and 120 BZS molecules to have a concentration of 25 mM following the highest concentration of our SAXS/SANS experiments.

We used PLUMED 2.8 ^53,54^ for the integrative approach using SAXS and SANS with resolution function.^55^ Using metainference,^39^ these simulations targeted both the SAXS and SANS curves simultaneously in the case of water and the SAXS curves only when salt was added. The use of SAXS and SANS simultaneously reduces the space of suitable configurations to describe both data, addressing the nonuniqueness problem. We follow a setup similar to that in our previous study.^11^ 20 *q* values in the experimental range were selected to describe the region around the peak in the *q* ≈ 0.1 Å^–1^ of the scattering curves, using independent Gaussian noise for each data point. The maximum effective uncertainty for each *q* was calculated as 0.7% of the total intensity at that point, while the minimum uncertainty was taken as 40% of this value. The maximum Monte Carlo displacement was defined to obtain approximately 50% acceptance of the uncertainty changes. As usual for these simulations, scaling and offset were also used to achieve the best agreement with the experiments and were sampled using Monte Carlo with a flat prior during the metainference simulations. All the input and output files can be accessed in the data repository that accompanies this work.^56^

## 3 Results and Discussion

### 3.1 Aggregation in Water

#### 3.1.1 ^1^H NMR and DOSY

NMR spectra in methanol-*d*_4_ or DMSO-*d*_6_ for BZS confirmed the symmetry of the molecule, with only two distinct singlet resonances for the triphenylene aromatic units. NMR experiments carried out in D_2_O evidenced a good solubility and a strong shielding effect on the chemical shift, particularly for the resonances of the aromatic CH of the triphenylene and the entire benzyl units with increasing concentration in solution (Figure S8, SI). Conversely, the hydrophilic butylsulfonate moieties were not greatly affected by the concentration. This effect is strongly indicative of aggregation phenomena driven by the hydrophobic effect, where molecules of BZS interact with each other, leaving the hydrophilic anionic side chains toward the bulk water and pushing the hydrophobic aromatic units close to each other. The critical aggregate concentration (CAC) was determined by plotting the chemical shift of the phenyl residue proton with respect to the inverse of the concentration by observing a marked change in the profile for BZS greater than 3.9 mM (see Figure S9). DOSY NMR experiments were carried out over a wide range of concentrations, observing in all cases for all resonances a monoexponential decay of the intensities with the gradient strength (Figure S14, SI). Through the diffusion coefficient values and the Stokes–Einstein equation, the average hydrodynamic radii of the species in solution showed a marked increase to 1.5 nm for solutions up to 2 mM, increasing to 2.5 nm for more concentrated solutions (Figure S16, SI). When the sizes of the solvated aggregates in solution and of the hydrophobic and hydrophilic units in BZS are compared, the emerging picture is that nanoassemblies in water were formed by a small number of molecules. This finding is likely to be ascribed to the presence of alternating residues of opposite polarities on the triphenylene units, which tends to frustrate the self-assembly process.

For the HS control molecule, lacking the hydrophobic alternating benzyl residues, the ^1^H NMR spectra in D_2_O at different concentrations showed higher shifts, particularly for the triphenylene CH residues with a CAC of 3.3 mM (Figure S13, SI), suggesting a pronounced π-stacking between units in water. DOSY experiments (Figure S17, SI) evidenced a multiexponential decay indicative of the formation of several types of aggregates in solution, with dimensions in the range of 1.2 nm, smaller compared to BZS and not much larger than the size of the single molecule.

Further evidence of BZS aggregation in water above the CAC was observed by plotting the fluorescence intensity as a function of the concentration. We observed a nonlinear profile with a maximum intensity of the band at 498 nm around 20 mM (see Figure S22, SI). This clearly suggests the formation of intermolecular π–π interactions between triphenylene units, which causes aggregation induced quenching of fluorescence.

#### 3.1.2 AFM

AFM analyses for aqueous samples of BZS deposited and dried on mica surfaces were performed to obtain qualitative information about the size and shape of the aggregates since measurements on dried samples do not reflect exactly the size of the aggregates present in aqueous solution. The images showed the presence of spherical particles with a radius of approximately 4–5 nm and a very narrow size distribution (Figure 4a). It is likely that the particles observed in the absence of water could be characterized by higher aggregation compared to samples in water, thus justifying the larger size observed with AFM in comparison to that observed with DOSY. Analogous analysis of the control HS sample did not display any tendency toward aggregation, in agreement with the results in solution.

**Figure 4:**
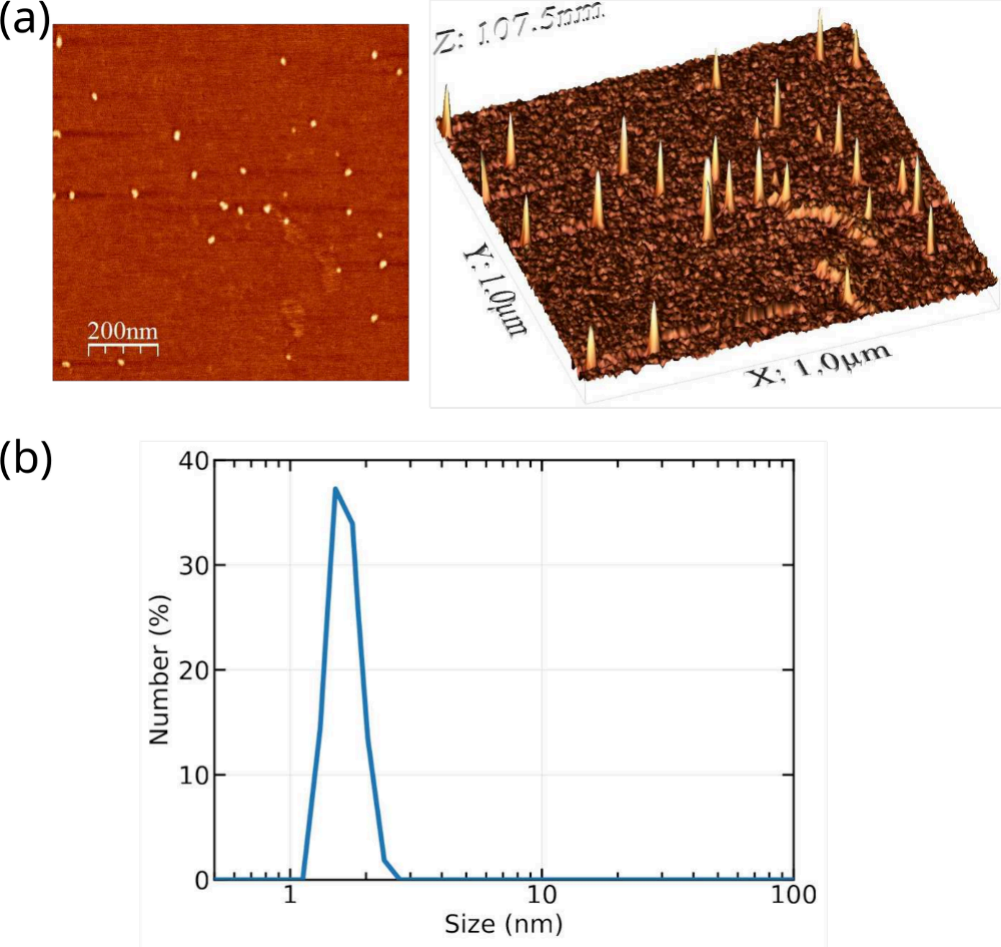
(a) AFM images for BZS and (b) DLS analysis of a 25 mM solution of BZS in water.

#### 3.1.3 DLS

In addition to AFM and DOSY, the aggregates were also investigated using DLS experiments, which were performed for a 25 mM solution of BZS in water. The results, displayed in Figure 4b, show one main distribution of particles with an average size of about 1.5–2.0 nm with a rather narrow distribution. The DLS results are then compatible with those found in the DOSY analyses.

#### 3.1.4 Small-Angle Scattering

The curves for the SAXS experiments performed at different concentrations are shown in Figure 5a. At low concentration (0.8 mM), the scattering intensity displays a steady featureless increase as *q* decreases. Upon increasing concentration, however, a peak at approximately *q* ≈ 10^–1^ Å^–1^ begins to appear, becoming fully visible at the highest measured concentration (25 mM). The apparent peak is a result of the decrease of scattering intensity at low *q*—impact of structure factor on the SAXS spectra—originating from the strong electrostatic repulsive interaction between molecules. This phenomenon can be seen more clearly when we normalize the scattering intensity by dividing it by concentration (Figure S27, SI). We observe that for concentrations *C*_BZS_ ≥ 3.1 mM, the structure factor plays a significant role, while scattering at high *q* maintains the same intensity. Thus, these findings do not suggest the existence of large assembled structures, possibly due to the unfavorable balance between electrostatic attractions and repulsions. To further investigate this effect, we performed SAXS experiments at the highest BZS concentration of 25 mM for increasing concentration of NaCl salt (0.1 M NaCl and 0.5 M NaCl solution). The results, shown in Figure 5b, confirm an increase in the scattering intensity at low *q* with increasing salt concentration, supporting the idea that electrostatics plays an important role in the self-assembly process. The overall intensity of the spectra shifts as a result of the difference in the solvent electron density, resulting in a lower contrast between the solvent and the BZS molecule. To minimize the impact of the contrast difference in the spectra, we normalized the scattering intensity and present the normalized data in Figure S28, SI. We do not observe significant differences between the normalized scattering pattern for the *q* values above 10^–1^ Å^–1^, and we notice that the structural factor disappears at higher salt concentrations. That indicates a weakening of repulsive electrostatic interactions as the ionic strength of the solution increases. Even at the highest concentrations, the data do not suggest that large aggregates are present.

**Figure 5:**
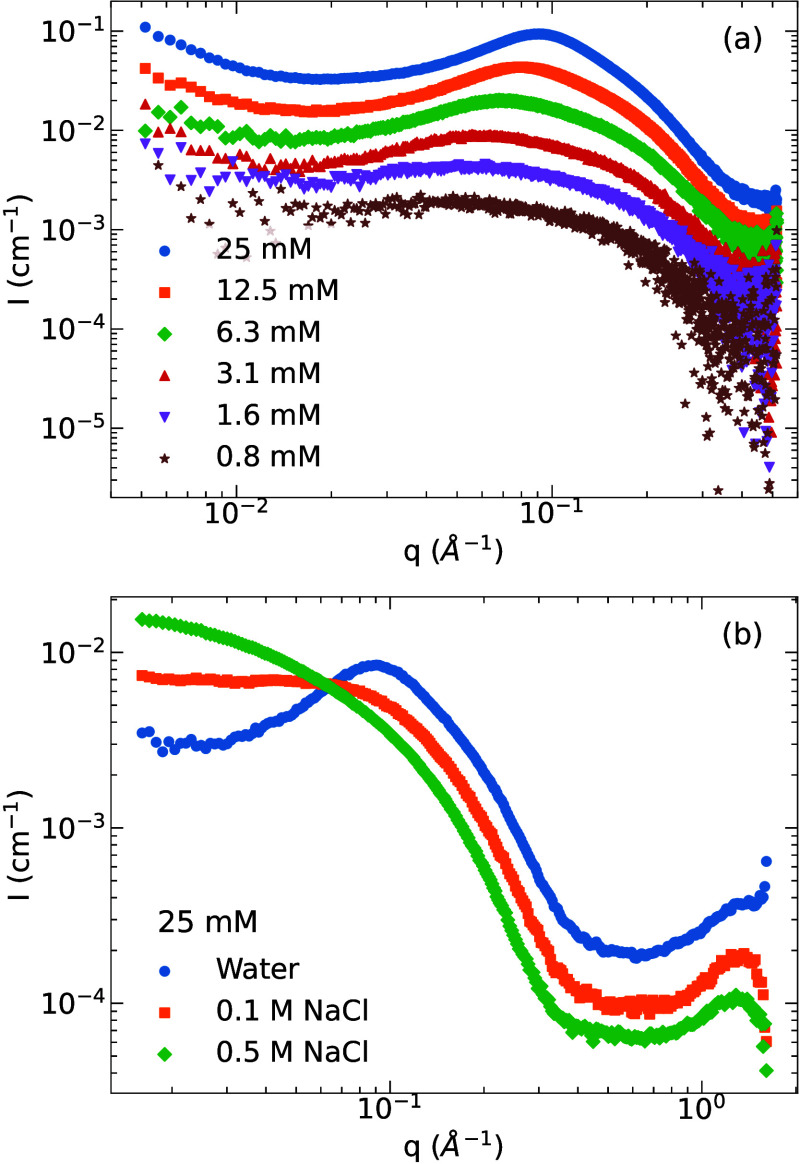
(a) SAXS curves for BZS solutions at different concentrations; data measured at BM29 synchrotron beamline. (b) SAXS curves for 25 mM BZS solutions in water, 0.1 M NaCl, and 0.5 M NaCl solution; data measured at ID02 synchrotron beamline.

It should be noted that the SAXS measurements reported in Figure 5b are for a concentration of 25 mM, largely above the CAC, and extend up to *q* = 1.6 Å^–1^ to also probe the microscopic organization of the assembled structure. These results are consistent with the results from the BM29 beamline, and the upturn at *q* ≥ 1 Å^–1^ hints at the formation of stacks in pure water. For triphenylene-substituted discotic liquid crystals in which there is π-stacking, the distance between the two monomers is typically around 3.4 Å,^57^ corresponding to *q* ≈ 1.9 Å^–1^. Therefore, the trend of increasing intensity may indicate the formation of stacks. However, the absence of the peak maximum in the examined range prevents us from determining the exact distance between potential stacks.

For the 25 mM concentration, we also performed SANS experiments to help characterize the aggregates (Figure 8a). One of the advantages of SANS is the different range of probed *q* values (∼0.001–1 nm^–1^ for SANS and ∼0.1–5 nm^–1^ for SAXS), thus allowing access to larger length scales. These results are consistent with those stemming from SAXS, having the *q* ≈ 0.1 Å^–1^ peak corresponding to interparticle distances.

Although the above experiments provide coherent evidence of aggregation, the actual organization and the driving force of the aggregation process remain unclear. Due to the shape of the BZS molecule, defining a form factor for model fitting using the SAXS and SANS results is challenging. To further investigate the shape of the aggregates, we resort to molecular dynamics simulations.

#### 3.1.5 All-Atom Simulations

The bulk of the evidence stemming from experimental results can be summarized as follows. The BZS molecule is composed of a rigid hydrophobic core of approximately 5 Å and by 6 alternating hydrophilic and hydrophobic tails having approximate lengths of 6 and 7 Å, respectively, hence providing a *C*_3_ symmetry. Note that the molecule is chiral, and hence, the two faces are not equivalent. In pure water, we find evidence of small aggregates (of the order of 2–3 nm) presumably resulting from a π-stacking with a possible additional twist to respect the *C*_3_ symmetry. The addition of salt is expected to induce further aggregation in view of the screened effect played by the salt on Coulomb repulsion between different BZS molecules. To confirm this putative scenario, we performed extensive numerical simulations at both all-atom and coarse-grained levels.

Figure 6a displays a representative snapshot of the all-atom simulation with water molecules removed for clarity. The snapshots also highlight a representative observed cluster formed by 4 BZS molecules stacked one on top of the other, with a clear twist of the hydrophilic moieties, which are all observed to be hydrogen bonded to water. It is further seen that these hydrogen bonds decrease with simulation time, likely reinforcing the BZS–BZS stacking over BZS–water contacts. The most stable conformation is found to be a tetramer rather than a trimer, which would be more consistent with the *C*_3_ symmetry. The reason can be traced back to the up–down symmetry breaking of the two faces. As a consequence, the 4mer-BZS is formed by four stacked units of repeated up–down–up–down conformations, which justifies the tetramer structure.

**Figure 6:**
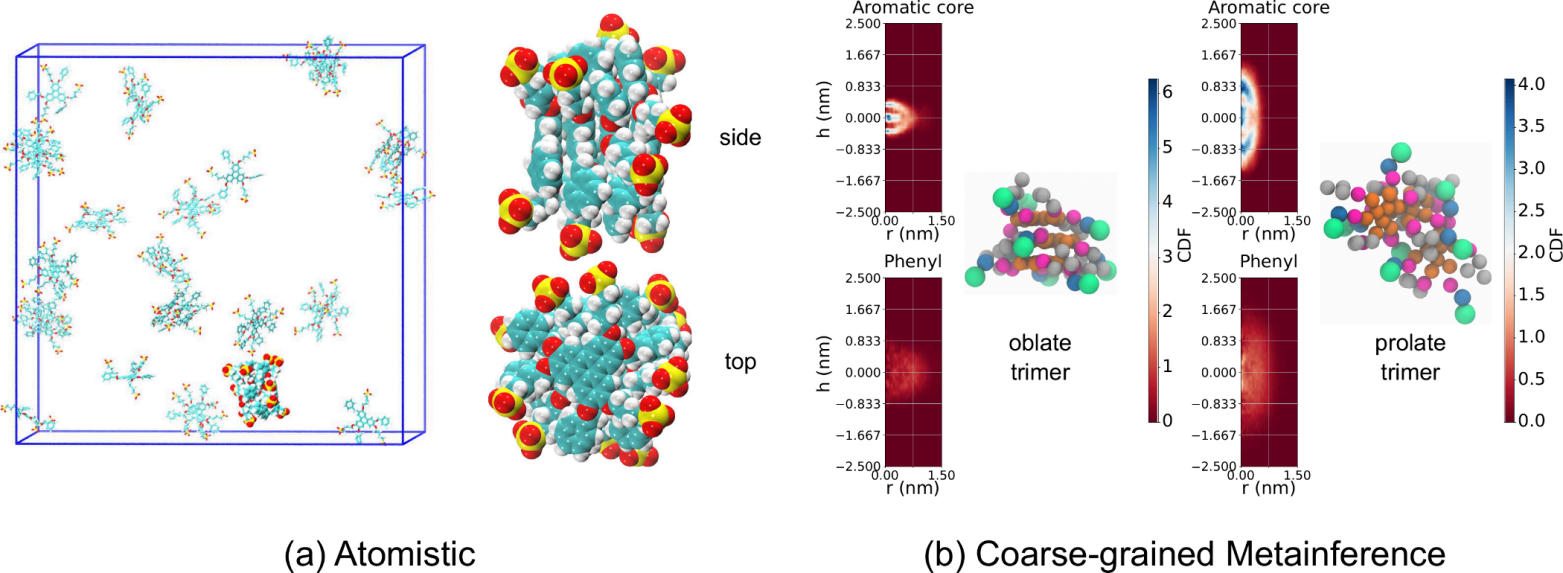
(a) Representative snapshot and close-up views of the largest 4mer-BZS identified in the all-atom simulation of BZS in water. (b) Cylindrical distribution function and representative snapshots of the trimers found in the coarse-grained metainference simulation of BZS in water. Water molecules are not shown for enhanced clarity.

Figure 7 reports different order parameters that support the formation of small aggregates. The total solvent-accessible surface area (SASA, Figure 7a) is found to gradually decrease with the simulation time, clearly indicating an aggregation process. Also, the radial distribution of the BZS–BZS center of mass (Figure 7b) indicates that this aggregate has dimensions of 6.5 Å, which is consistent with the characteristic distance between two stacked BZS molecules. It should be noted that this distance is also compatible with the threshold cutoff minimum distance of about 4.2 Å within which two BZS moieties can approach each other found in the potential of mean force (PMF) curve (see Figure S37, SI). This is further confirmed by measuring the separation distance between adjacent nearest-neighbor oxygen atoms of the aliphatic oxy-butylsulfonate tails in two centromer BZS moieties (Figure 7c). After a transient of approximately 15 ns, all these different neighbor atoms were found to converge to a distance of ∼1 nm (Figure 7c). Finally, Figure 7d displays the cluster size distribution that confirms that only clusters up to 4mers are observed. Note that this cluster distribution was performed throughout the trajectory, including equilibration, thus explaining the subdominance of the tetramer. Different tests were performed to confirm the stability of these 4mer configurations (see Section S3.4, SI). The configurations for the tests included sandwich-like, parallel-offset, and side-to-side type arrangements in a cubic simulation box (∼6 × 6 × 6 nm^3^). In all cases (Figure S38, SI), the tetramer units remained stable, and no additional signs of growth in aggregation were found, confirming the stability of the 4mers.

**Figure 7:**
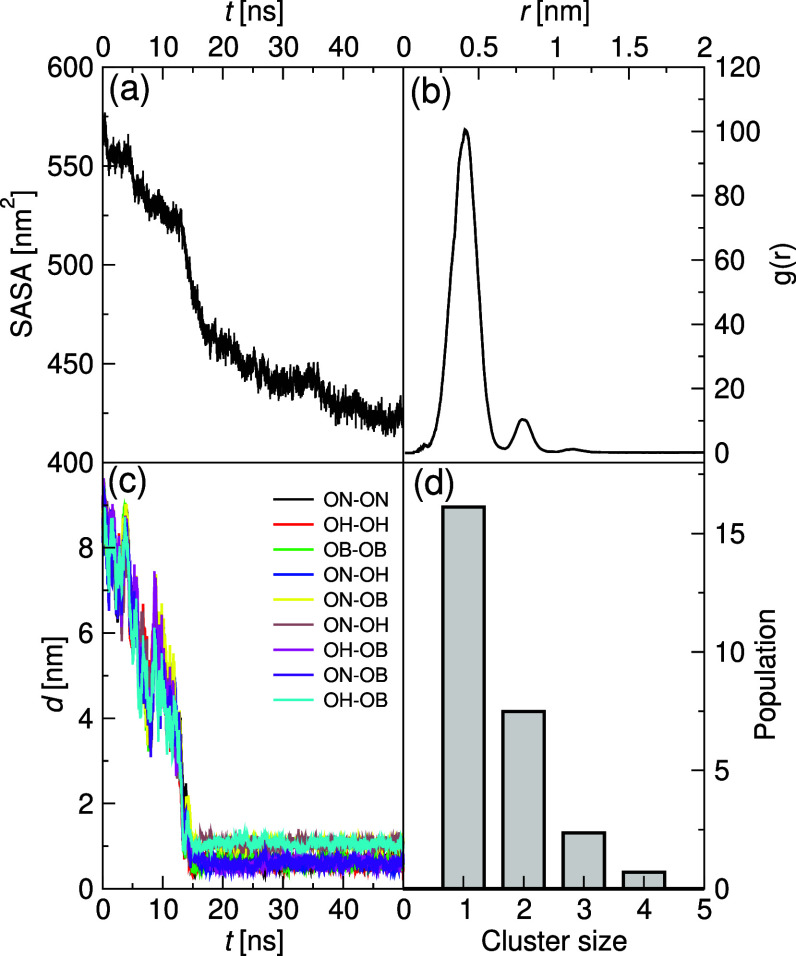
Order parameters for the all-atoms simulations of BZS self-assembly process. (a) The time-based total solvent-accessible surface area of BZS entities; (b) the BZS–BZS center of mass pair radial distribution function; (c) the separation distance between adjacent nearest-neighbor oxygen atoms of aliphatic oxy-butylsulfonate tails in 2 centromer BZS moieties as a function of time; and (d) the cluster size distribution.

We further tested the stability of the tetramer against possible competing aggregation induced by the presence of ADL molecules, which may be expected to destabilize the formation of the tetrameric aggregates. We did not observe any sign of tetramer dislocation, which is consistent with previous findings. However, while no specific ADL–ADL or ADL–BZS aggregation patterns could be seen, clear evidence of BZS–BZS aggregation growth was observed (see Figures S39 and S40, SI).

#### 3.1.6 Metainference Simulations

The unbiased MD simulations using Martini 3 were run for 600 ns and favored the formation of relatively large polydisperse aggregates using the 120 BZS molecules in the simulation box to form on average 3.7 aggregates with varying sizes (Figure S29, SI). These results are inconsistent with the all-atom simulations and may be due to a shortcoming in the parametrization of the coarse-grained force field for the description of BZS. Using different bead types provided different aggregates, with more stacking or slight changes in the aggregate size distribution, but in general, large aggregates were observed. Moreover, the simulated SAXS and SANS intensities obtained with snapshots from these simulations were in poor agreement with the experimental curves.

A better agreement with the experiment and all-atom simulations is recovered by performing metainference simulations targeting SAXS and SANS simultaneously. Since we are more interested in the structure of the aggregates, we focus on the intermediate/high *q* region, where the structure factor does not play a major role. These simulations were run for 150 ns, and the last 80 ns were used for the analysis. In Figure 8 we show the computed spectra obtained with these simulations. The average scattering curve computed with metainference, shown in orange, is in very good agreement with the experimental spectra, showing the same features. The thin lines in the figure represent the spectra from single snapshots and indicate that the overall spectrum is composed of relatively similar configurations. Using the metainference effective uncertainty parameters, we obtain a χ^2^ of 6.93 for SANS and 0.17 for SAXS.

**Figure 8:**
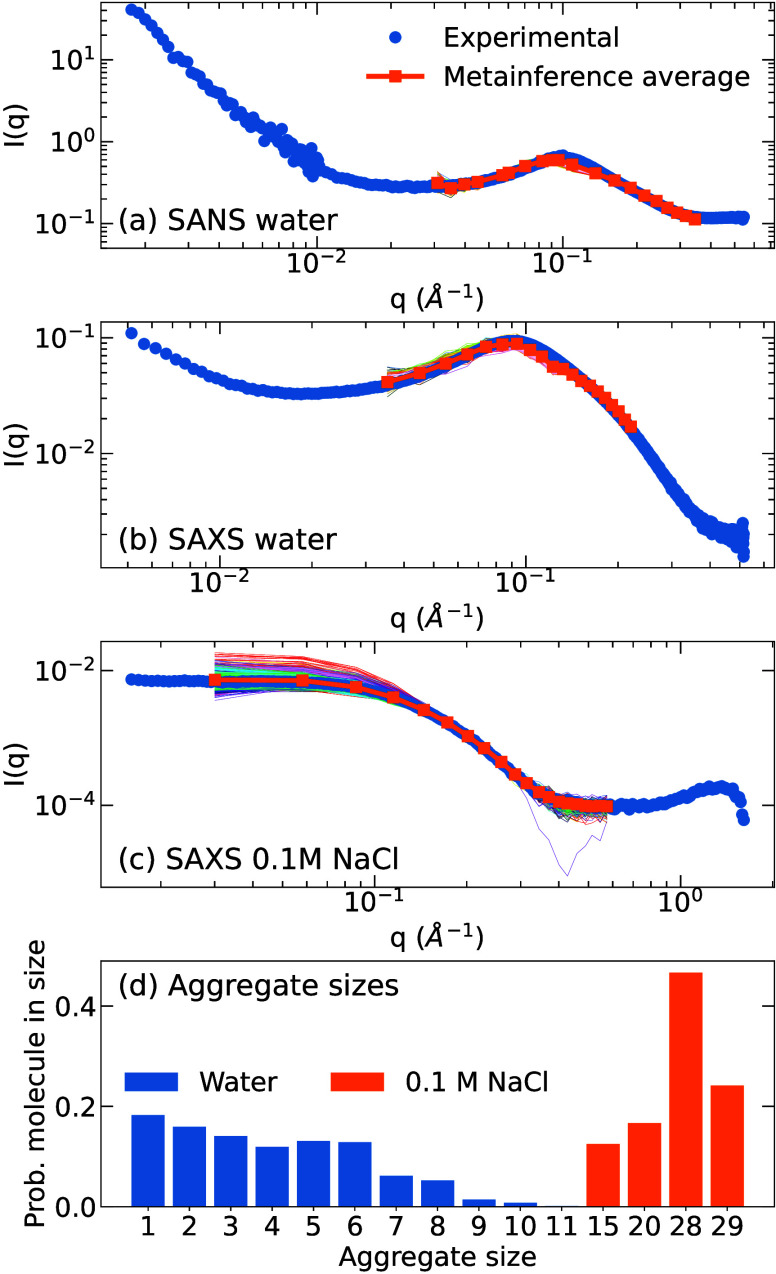
SAS of BZS from the metainference simulations and aggregate size distribution. The results are for 25 mM BZS in water (SANS in panel (a) and SAXS in (b)) and 0.1 M NaCl (SAXS in (c)). The colored thin lines represent the computed spectrum for individual snapshots. (d) The probability of a molecule belonging to an aggregate of a given size.

The metainference bias is able to overcome the shortcomings in the coarse-grained force field, thus providing evidence of the predominance of the smaller stacked aggregates, in agreement with all-atom simulations, albeit additional types of aggregates are still observed. Some snapshots of the sampled configurations can be seen in Figure S32 in the SI, from which one can identify the stacking. Most stacks are composed of up to 4 molecules, while the larger aggregates are the result, in general, of a less organized packing with the merging of smaller aggregates.

A more detailed analysis of the aggregation in pure water is shown in Figure 8d. Almost 20% of the molecules are dissolved and do not aggregate, while the remaining 80% are distributed in aggregates with up to 8 molecules, with aggregates composed of more than 6 molecules being rare. The position of the functional groups is connected to the angles and dihedrals (see Figure S34, SI), pointing to more closed dihedrals for the phenyl groups, meaning that they tend to stay closer to the triphenylene core, and more open dihedrals for the oxy-butylsulfonate tails. Such distributions can be understood by considering that the charged groups try to be exposed to the surface, allowing interactions with the solvent and counterions.

We also separated the aggregates by shape and analyzed the cylindrical distribution function (CDF) for aggregates of some sizes. The CDF is a density map that shows how each bead type is distributed in space after the alignment of the aggregate’s principal moments of inertia given by the eigendirections associated with the eigenvalues of the moment of inertia tensor. For this analysis, we label the aggregates as prolate, oblate, or nearly spherical depending on the relative ratios of the eigenvalues of the moment of the inertia tensor. More details on this type of analysis are given in a previous work.^11^ The results for the 3mer are shown in Figure 6b (results for other sizes in Figure S33, SI). Most structures are labeled prolate (about 80%), but a significant amount of oblate structures (about 15%) are also present for all sizes. For the dimer and trimer, the oblate structures correspond to the perfectly stacked configurations. However, we see from the aromatic-core-bead density of Figure S33, SI that some loose stacking is preferred, even if the planar aromatic regions are not completely parallel. The CDFs for the phenyl beads show that, as a result of its hydrophobicity, these groups tend to stay close to the core, while the sulfonate beads are mostly exposed to water. This creates a complex structure with stacking, but one that resembles core–shell aggregates due to the segregation of the flexible hydrophobic and hydrophilic moieties.

### 3.2 Self-Assembly of BZS Varying the Ionic Strength

We also performed SAXS metainference simulations with 0.1 M added NaCl to investigate the screening effects of the added salt on the aggregates. In this case, the simulations only targeted the SAXS curve and were performed for 200 ns, with the last 100 ns used for the analysis. We obtained a very good agreement for the SAXS curve, with χ^2^ = 2 × 10^–5^.

The aggregation analysis for the 0.1 M NaCl solution is shown in Figure 8d. It is evident that the presence of salt favors the formation of larger aggregates. Visually inspecting the structures, we see that the stacks are less prominent, and instead, relatively longer cylindrical aggregates are observed (Figure S32c,d, SI). These results are consistent with all-atom simulations performed on the same system (see Section S3.3, SI).

We also analyzed the CDFs and perpendicular RDFs for these aggregates, as presented in Figure 9. The analysis reveals a preference for the triphenylene group to concentrate at the top and bottom of the hydrophobic core. In the case of the 28mer, this leads to a dumbbell-shaped aggregate, almost splitting it in half. However, the phenyl groups tend to stay in the center and help to compose the hydrophobic core of the aggregates. Similarly to the prolate aggregates formed in the water solution without NaCl, the hydrophilic groups of the sulfonates tend to stay mostly in the shell, so the aggregate has a core–shell-like structure.

**Figure 9:**
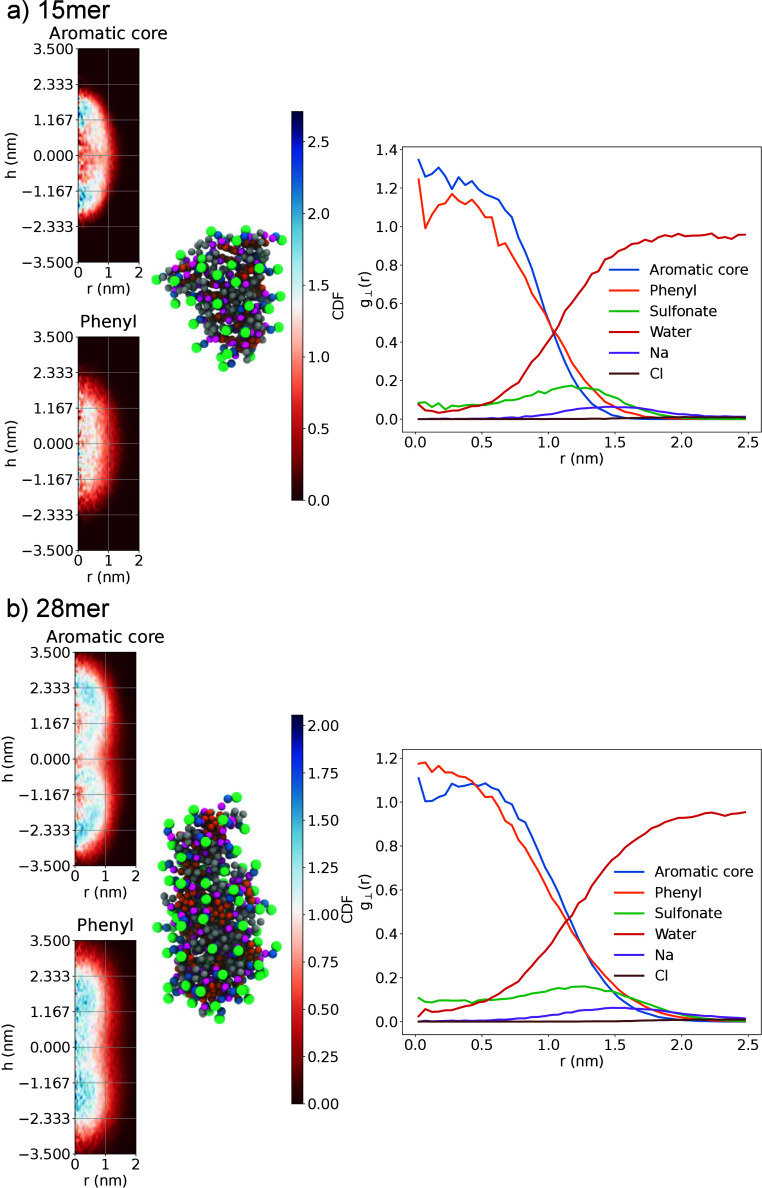
CDFs (left) and perpendicular RDFs (right) for two aggregate sizes from the metainference BZS simulation in 0.1 M NaCl. The perpendicular RDFs are the integration of the CDFs over the symmetry axis. (a) Densities and structure for the 15mer. (b) Densities and structure for the 28mer. The axis of symmetry is aligned along the vertical. The bead color of the example configurations follow the bead types of Figure 3.

The RDFs allow for a better identification of the overlapping densities. Despite having an almost core–shell structure, the aggregates have some intermixing between the hydrophobic and hydrophilic moieties, including some water and Na^+^ penetration. As expected, there is also layering of Na^+^ around the aggregates, while the Cl^–^ ions are uniformly distributed in the solution.

A comparison of distribution functions with and without added NaCl is not straightforward because the aggregate size distribution does not overlap. However, we can compare the effects of the charges on the intramolecular interactions. These results are shown in the SI, where we plot the distributions of angles and dihedrals under both conditions (Figure S34, SI). We observed that the screened electrostatic interactions allow the charged groups to move more freely. This is clear from the dihedral angle distribution of the oxy-butylsulfonate tails. The increased flexibility can be interpreted, in terms of conventional packing parameter quantities, as a reduction in the effective surface area of the polar head. This is due to the decreased charge-repulsion between neighboring SO_3_^–^ groups. As the sulfonate groups are free to move toward the solvent and the Na^+^ layer, more space is available in the core region for more amphiphiles to compose the aggregate. In addition, the increased flexibility competes with the organization in the assemblies, which in pure water have alternating hydrophobic and hydrophilic groups. Hence, the aggregates in NaCl are not only larger but also more disordered.

A similar effect of the ionic strength, significantly enhancing the aggregation number and modifying the morphology of the micellar structure, was previously reported for sodium dodecyl sulfate (SDS).^10^ Also in that case, interference of the mobile positive ions with the negatively charged head of the surfactant could explain the experimentally observed structural changes. Thus, these most recent findings on BZS indicate a general salt-mediated mechanism that controls aggregation in charged surfactants. Unlike other cases, such as SDS or lipopeptides, where the hydrophobic moiety of the surfactant is spatially distinct from the charged/hydrophilic part, for the more scrambled topology of BZS, with intermixed hydrophilic and hydrophobic regions, charge-screening becomes the crucial ingredient promoting aggregation. The possibility of controlling micellar self-assembly by acting on the local ionic strength may be exploited in technological setups aiming at targeted sequestration/releasing of encapsulated compounds as a response to environmental changes.

## 4 Conclusions

In this work, we report a broad experimental and computational study of BZS, a triphenylene-based frustrated amphiphile with alternating hydrophobic and hydrophilic groups. In addition to small-angle scattering experiments with X-rays and neutrons, we produced molecular models displaying very good agreement with those signals by combining coarse-grained MD simulations with metainference.

Despite the complex shape of the molecule, the organization of the larger micelles observed in saline solutions resembles that of the conventional core–shell model. Specifically, the phenyl and triphenylene groups accumulate in the interior, while the sulfonate groups point toward the solvent. Expectedly, the Na^+^ ions bind weakly to the surface of micelles, screening the negatively charged sulfonate groups and thus facilitating aggregation. The structural identification of these aggregates was only possible by multiscale modeling including coarse-grained metainference simulations targeting the small-angle scattering data and all-atom simulations. The complex shape of the BZS molecule, in fact, makes it challenging to predict the aggregate shape and model the data fitting form factors, especially for the type of stacked structures we observed.

In agreement with previously studied charged surfactants, our data on BZS highlight the critical impact of the ionic strength of the solution on the size and shape of the micellar aggregates present in the experimental samples. In this particular case, our data reported small dimeric-hexameric assemblies observed in the sample that are stabilized by the formation of π-stacking interactions involving the triphenylene core when BZS is dissolved in pure water. On the contrary, larger and less organized assemblies, with an aggregation number fluctuating between 15 and 29 molecules, are present in aqueous solution with 0.1 M NaCl. The stability of the aggregates found by the integrative modeling was further validated by complementary molecular dynamics simulations with all-atom resolution. These results reinforce the role of salt in determining the structural properties of charged micelles and suggest the possibility of exploiting environmental control as a route for targeted molecular trafficking in (bio)­chemical processes.

## Supplementary Material



## Data Availability

The small-angle X-ray scattering data for this article are available at the European Synchrotron Radiation Facility repository at https://doi.esrf.fr/10.15151/ESRF-ES-924497609 and https://doi.esrf.fr/10.15151/ESRF-ES-1078543878. The simulation data are deposited on NIRD: 10.11582/2025.5rw59tyu.

## Notes

Synthesis details for BZS and HS, additional experimental results (mass spectra, NMR spectra, UV–vis and fluorescence spectra, and SAXS/SANS spectra), and additional simulation results (aggregation analysis for coarse-grained simulations, conformational differences in water and NaCl solution, BZS simulation with salt, atomistic PMF, and BZS aggregates stabilized by ADL) (PDF)
