# Supplementary material for: Self-Assembly of Unconventional Triphenylene-Based Frustrated Amphiphile in Solution
Source: Langmuir. 2026 Feb 6;42(6):4507–17. doi: 10.1021/acs.langmuir.5c05203 (PMC12922186; doi:10.1021/acs.langmuir.5c05203)
Supplement: Supplementary file 1 [file la5c05203_si_001.pdf]

Supporting Information: Self-assembly of unconventional triphenylene-based frustrated amphiphile in solution

Henrique Musseli Cezar,[*,†,‡] Giacomo Berton,[¶] Tommaso Lorenzetto,[¶] Sandro Zorzi,[¶,§] Cedrix J. Dongmo Foumthuim,[¶,‖] Szymon Mikołaj Szostak,[‡] Pablo Ballester,[⊥,#] Claudia Mondelli,[@] Ralf Schweins,[△] Viviana Cristiglio,[△] Fabrizio Fabris,[¶] Reidar Lund,[†,‡,∇] Alessandro Scarso,[*,¶] Achille Giacometti,[*,¶,††] and Michele Cascella[*,†,‡]

†*Hylleraas Centre for Quantum Molecular Sciences, University of Oslo, PO Box 1033 Blindern, 0315 Oslo, Norway*

‡ *Department of Chemistry, University of Oslo, PO Box 1033 Blindern, 0315 Oslo, Norway*

¶*Dipartimento di Scienze Molecolari e Nanosistemi, Università Ca' Foscari Venezia, via Torino 155, 30172, Venezia, Italy*

§*Fondazione Bruno Kessler – FBK, Via Sommarive 18, 38123 Povo (TN), Italy*

‖ *Department of Chemical Sciences, University of Padova, via Marzolo 1, 35131 Padova, Italy*

⊥*Institute of Chemical Research of Catalonia (ICIQ), Barcelona Institute of Science and Technology, Avinguda Països Catalans 16, Tarragona 43007, Spain*

#*Catalan Institution for Research and Advanced Studies (ICREA), Passeig Lluis Companys 23, Barcelona 08010, Spain*

@*CNR-IOM, Institut Laue Langevin 71 Avenue des Martyrs 38042 Grenoble Cedex 9, France*

△*Institut Laue Langevin 71 Avenue des Martyrs 38042 Grenoble Cedex 9, France*

∇*Donostia International Physics Centre (DIPC), Manuel Lardizabal Ibilbidea, 4, 20018 Donostia, Gipuzkoa, Spain*

††*European Centre for Living Technology (ECLT) Ca' Bottacin, Dorsoduro 3911, 30123-Venice, Italy*

E-mail: h.m.cezar@kjemi.uio.no; alesca@unive.it; achille.giacometti@unive.it; michele.cascella@kjemi.uio.no

Contents

S1 Synthesis details **S-7**

S1.1 BZS S-7

S1.2 HS S-7

S2 Experimental results **S-8**

S2.1 Mass spectra S-8

S2.1.1 BZS S-8

Figure S1 - Mass spectra of BZS - Part 1 . . . . S-8

Figure S2 - Mass spectra of BZS - Part 2 . . . . S-9

Figure S3 - Mass spectra of BZS - Part 3 . . . . S-9

S2.1.2 HS S-10

Figure S4 - Mass spectra of HS - Part 1 . . . . S-10

Figure S5 - Mass spectra of HS - Part 2 . . . . S-11

S2.2 NMR S-11

S2.2.1 BZS S-11

Figure S6 - ^{1}H NMR spectrum of BZS S-12

Figure S7 - ^{1}H NMR spectra of BZS S-12

Figure S8 - Summary of ^{1}H NMR spectra of BZS in D_2O as a function of the concentration.S-13

Figure S9 - Plot of the aromatic CH chemical shift of BZS with respect to the inverse of the concentration. The two lines used to calculate the critical aggregation concentration are reported join at 3.9 mM. S-14

S2.2.2 HS S-14

Figure S10 - ^{13}C-NMR spectrum of HS in dsmo-d_6. . . . . S-15

Figure S11 - ^{1}H NMR spectra of HS S-15

Figure S12 - Summary of ^{1}H NMR spectra of HS in D_2O as a function of the concentration.S-16

Figure S13 - Plot of the aromatic CH chemical shift of HS with respect to the inverse of the concentration. The two lines used to calculate the critical aggregation concentration are reported join at 3.3 mM. S-17
S2.3 DOSY NMR S-17
S2.3.1 BZS S-17
Figure S14 - Pseudo2D-DOSY NMR mono-exponential decay of BZS benzyl signals in D_2O at different concentrations. S-18
Table S1 - Diffusion coefficients (D) of the two rounds of DOSY analysis of BZS in D_2O at different concentrations. S-18
Figure S15 - Diffusion coefficient of BZS as a function of concentration. S-19
Table S2 - Hydrodynamic radius of the two rounds of DOSY analysis of BZS in D_2O at different concentrations. S-19
Figure S16 - Hydrodynamic radius of BZS as a function of concentration. S-20
S2.3.2 HS S-20
Table S3 - Diffusion coefficients (D) of the two rounds of DOSY analysis of HS in D_2O at different concentrations. S-20
Figure S17 - Pseudo2D-DOSY NMR mono-exponential decay of HS triphenylenic signals in D_2O at different concentrations. S-21
Figure S18 - Diffusion coefficient of HS as a function of concentration. S-21
Table S4 - Hydrodynamic radius of the two rounds of DOSY analysis of HS in D_2O at different concentrations. S-22
Figure S19 - Hydrodynamic radius of HS as a function of concentration. S-22
S2.4 UV-vis and fluorescence S-23
Figure S20 - UV-Vis spectra of BZS in water at different concentrations at 297 K. . . . S-23
Figure S21 - Plot of Absorbance vs. concentration for BZS in water. S-23
Figure S22 - Emission spectra of BZS in water at different concentrations at 297 K. Excitation wavelength 425 nm. S-24
Figure S23 - Plot of Fluorescence intensity vs. concentration for BZS in water. S-24
S2.5 Small-angle scattering S-25

S2.5.1 Model fitting S-25
Table S5 - Fit results for the BZS 25 mM solution in D2O, using the CSSxHmsa model. S-25
Figure S24 - SANS data (green triangles) and the best fit (black line) for the sample BZS 0.25 mM. S-26
Figure S25 - Kratky plot of the BZS 0.25 mM sample. S-27
Table S6 - Fit results for the BZS 25 mM solution in D_2O, using the stacked disk model. S-27
Figure S26 - SANS data (green triangles) and the fit (black line) using the stacked disk model for the sample BZS 0.25 mM. S-28
S2.5.2 SAXS additional plots S-28
Figure S27 - SAXS curves for BZS solutions, normalized by dividing the intensity by solution concentration S-29
Figure S28 - SAXS curves for BZS solutions, normalized to have the same relative contrast between BZS and solvent S-30

S3 Simulation results S-31

S3.1 Aggregation analysis for coarse grained simulations S-31
Figure S29 - Aggregate analysis from the unbiased Martini 3 simulations trajectories in water: (a) number of detected aggregates for trajectory frames; (b) average number of aggregates with given number of BZS molecules per snapshot; (c) probability of finding aggregate of given size in each snapshot; (d) probability of having a BZS molecule belonging to an aggregate of a given size. S-32
Figure S30 - Aggregate analysis from the Metainference simulations trajectories in water: (a) number of detected aggregates for trajectory frames; (b) average number of aggregates with given number of BZS molecules per snapshot; (c) probability of finding aggregate of given size in each snapshot; (d) probability of having a BZS molecule belonging to an aggregate of a given size. S-32

Figure S31 - Aggregate analysis from the Metainference simulations trajectories in 0.1 M NaCl solution: (a) number of detected aggregates for trajectory frames; (b) average number of aggregates with given number of BZS molecules per snapshot; (c) probability of finding aggregate of given size in each snapshot; (d) probability of having a BZS molecule belonging to an aggregate of a given size. S-33
Figure S32 - Example snapshots from the coarse-grained Metainference simulations. (a) and (c) the beads representing the polycyclic core, colored by molecule; and (b) and (d) all BZS beads colored by aggregate. The snapshots in (a) and (b) are from the water simulations, while the snapshots in (c) and (d) are for the simulations in 0.1 M NaCl solution. Water and ion beads were removed from the visualization for clarity. S-33
Figure S33 - CDFs and example snapshot for selected sizes from the Metainference BZS simulation in pure water. a) oblate aggregates for the dimer and trimer. b) prolate aggregates for the dimer, trimer and *8mer*. The axis of symmetry are aligned along the vertical. The bead color of the example configurations follow the bead types of Figure 3b of the main text. S-34
S3.2 Conformational differences in water and NaCl solution S-35
Figure S34 - Selected angles and dihedral angles distribution sampled in the Metainference simulations in water and 0.1 M NaCl solution. Labels for the angles are given in the atomic structure and represent angles of the coarse-grained structure (displayed in semi-transparent grey). The labels are separated for the phenyl and sulfonate groups. Vertical dashed lines for the angles represent the average. S-35
S3.3 BZS simulation with salt S-35

Figure S35 - Order parameters for all-atom simulations of BZS self-assembly process in pure water (black) and with 0.1M NaCl (red). (a) The time-based total solvent accessible surface area of BZS entities; (b) The cluster size distribution of BZS aggregate in simulation with salt and in pure water (in the inset); (c) The BZS-BZS center of mass pair radial distribution function; (d) Na^+-SO_3^{2-} pair radial distribution function. S-36

Figure S36 - Representative snapshot and close-up view of the largest 7mer-BZS identified in all-atom simulation of BZS in water with 0.1M NaCl. S-36

S3.4 Atomistic Potential of Mean Force S-37

Figure S37 - Potential of Mean Force (PMF) between BZS moieties as a function of their COM separation distance in pure water (black) and with 0.1M NaCl (red). S-37

Figure S38 - Overview of the initial starting configurations of BZS stacked assemblies used to further probe the stability of the tetrameric order arrangement corresponding to sandwich-like setup (a), parallel offset arrangement (b), and side-to-side display (c). BZS moieties are shown in licorice representations while solvent and ions are omitted for clarity. S-38

S3.5 BZS aggregates stabilized by ADL S-39

Figure S39 - (a) Overview of the final simulation (1 µs) box with the two largest BZS aggregates highlighted. ADL units are also shown as blue van der Waals spheres while interacting with BZS moieties. No signs of ADL self-assembly are reported. (b) The close-up views of the largest stable cluster *16mer*-BZS identified in the simulation is shown in two different representations with the central hydrophobic patch of BZS highlighted in the second view. . . . S-39

Figure S40 - (a) Atomistic simulation results of BZS/ADL model system. The time-based total accessible surface area of BZS entities. (b) The BZS-BZS center of mass pair radial distribution function. (c) The separation distance between adjacent nearest neighbour oxygen atoms of aliphatic oxy-butylsulfonate tails in the 2 centro-mers BZS moieties as a function of time. (d) The cluster size distribution. S-41

S1 Synthesis details

S1.1 BZS

In a 25 mL round-bottomed flask, a suspension of 3,7,11-tris(benzyloxy)triphenylene-2,6,10-triol (0.64 mmol), butan sultone (0.59 mL, 0.78 g, 5.8 mmol), and K_2CO_3 (1.0 g, 7.2 mmol) in of acetone (8 mL) was refluxed under inert atmosphere for 72 h. The resulting mixture was diluted in 10 mL of absolute ethanol, filtered, washed with absolute ethanol (3×10 mL) and dried under vacuum. The resulting solid was dissolved in 20 mL of water and passed through a wet Amberilite IR 120 H^+ form column (d = 2 cm, h = 40 cm). The recovered pale-yellow acidic solution was carefully titrated with 0.1 M and 0.01 M aqueous NaOH to pH 7.0 with the aid of a pH-meter. The neutral solution was frozen in liquid nitrogen and lyophilized, affording a fluffy white solid.

Sodium 4,4',4"-((3,7,11-tris(benzyloxy)triphenylene-2,6,10-triyl)tris(oxy))tris(butane-1-sulfonate) (BZS). 94 % yield. M.p. 230 °C (dec.). ^{1}H NMR (400 MHz): (DMSO-d_6) δ 8.01 (3H, s), 7.85 (3H, s), 7.65-7.60 (6H, set of m), 7.43 (6H, t, J = 7.8 Hz), 7.35-7.29 (3H, set of m), 5.43 (6H, s), 4.24 (6H, t, J = 5.6 Hz), 2.58 (6H, t, J = 7.6 Hz), 2.01-1.83 (12H, set of m). ^{13}C NMR (100 MHz): δ 149.1, 148.1, 138.1, 128.9, 128.1, 128.0, 123.4, 122.9, 108.4, 106.8, 70.7, 68.9, 51.7, 28.8, 22.6. IR (KBr): ν 3428, 2917, 2851, 1618, 1514, 1454, 1435, 1382, 1264, 1201, 1185, 1168, 1156, 1041,1023, 856, 743, 729, 696, 614, 598 cm^{-1}. HRMS (ESI-Orbitrap): calcd. for $C_{51}H_{51}Na_2O_{15}S_3$ [M^-] 1045.2191; found: 1045.2258; calcd. for $C_{51}H_{51}NaO_{15}S_3$ [M^{2-}] 511.1149; found: 511.1151; calcd. for $C_{51}H_{51}O_{15}S_3$ [M^{3-}] 333.0802; found: 333.0804.

S1.2 HS

Sodium 4,4',4"-((3,7,11-trihydroxytriphenylene-2,6,10-triyl)tris(oxy))tris(butane-1-sulfonate) (HS). In a 50 mL round bottomed flask, BZS (0.150 g, 0.14 mmol) was dissolved in MeOH (26 mL). Pd/C 10 % (50 mg) was added and the suspension was putted under hydrogen atmosphere (1 bar, balloon) and stirred at room temperature for 18 h. The resulting sus-

pension was filtered on a celite plug and washed with MeOH. The resulting solution was dried in vacuum, to afford the product as a grey solid (0.100 g, 0.13 mmol, 90 % yield). M.p. °C (dec.). ^{1}H NMR (400 MHz): (DMSO-d6) δ 9.25 (3H, s), 7.77 (3H, s), 7.68 (3H, s), 4.12 (6H, t, J = 5.7 Hz), 2.56 (6H, t, J = 7.4 Hz), 1.96 - 1.86 (6H, m), 1.86 - 1.77 (6H, m). HRMS (ESI-Orbitrap): calcd. for $C_{30}H_{33}NaO_{15}S_3$ [M_2^-] 376.0445; found: 376.0441; calcd. for $C_{30}H_{33}O_{15}S_3$ [M_3^-] 243.0333; found: 243.0337.

S2 Experimental results

S2.1 Mass spectra

S2.1.1 BZS

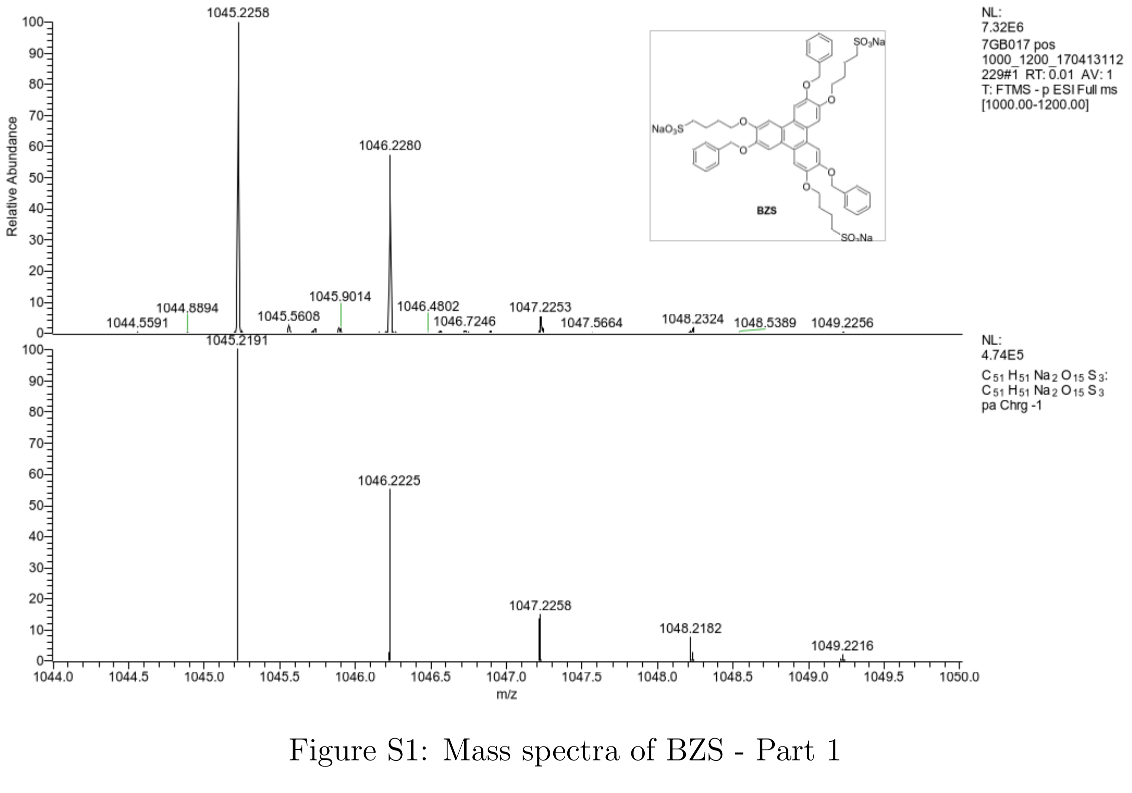

Figure S1: Mass spectra of BZS - Part 1

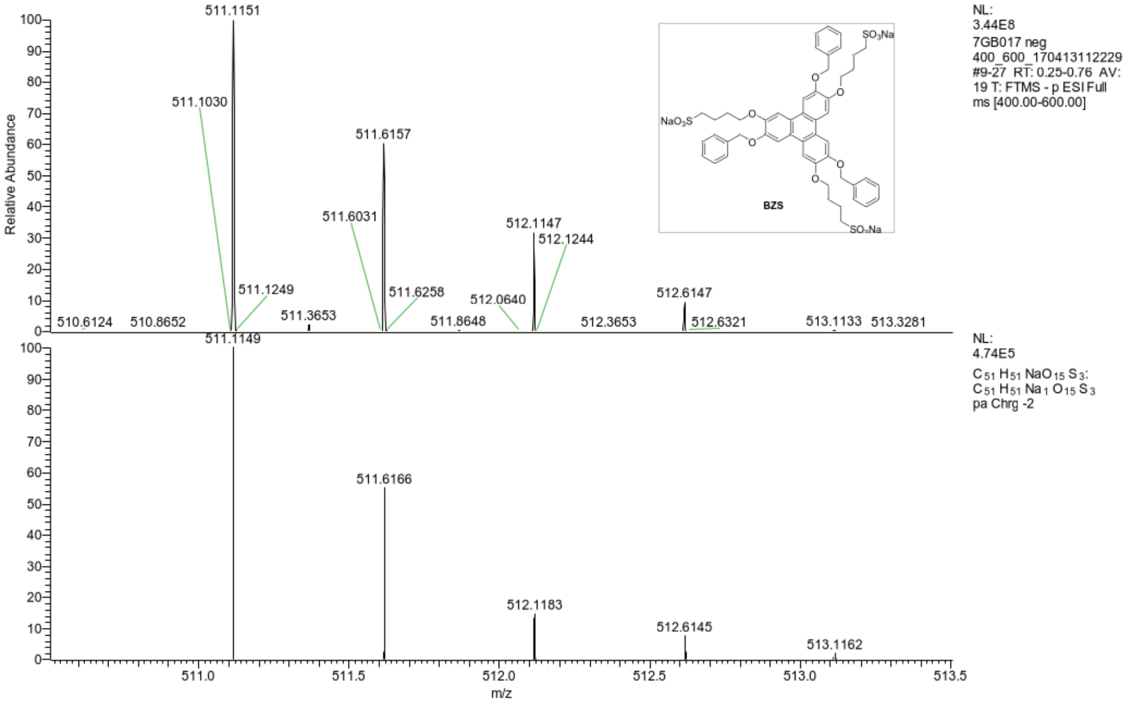

Figure S2: Mass spectra of BZS - Part 2

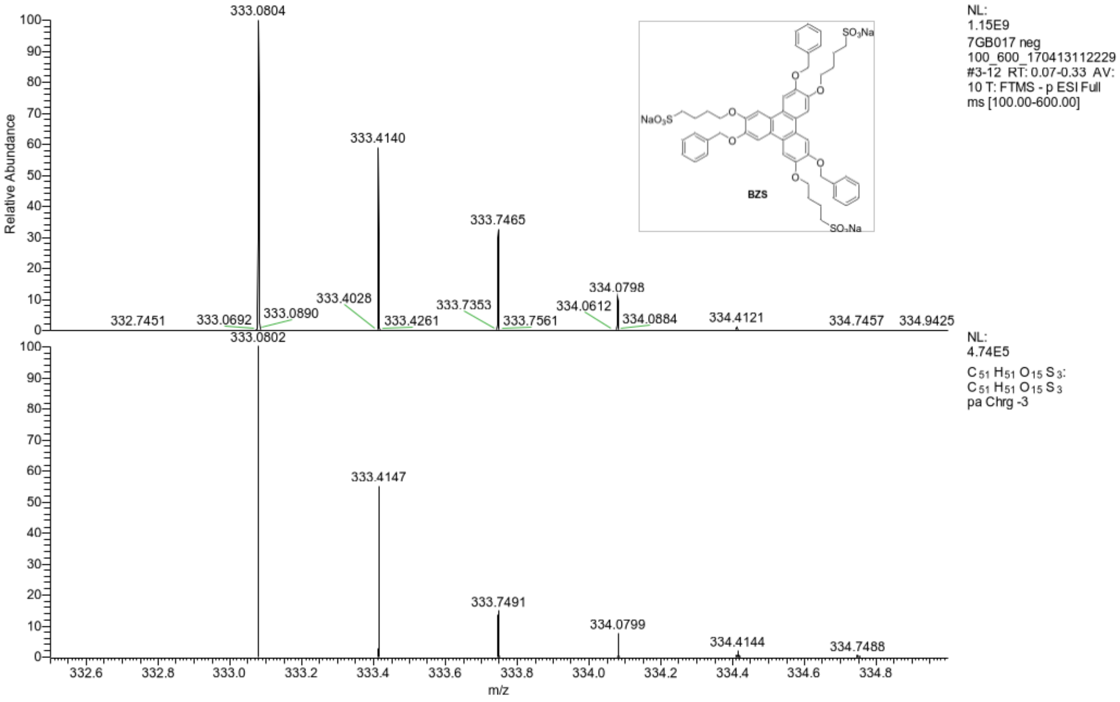

Figure S3: Mass spectra of BZS - Part 3

S2.1.2 HS

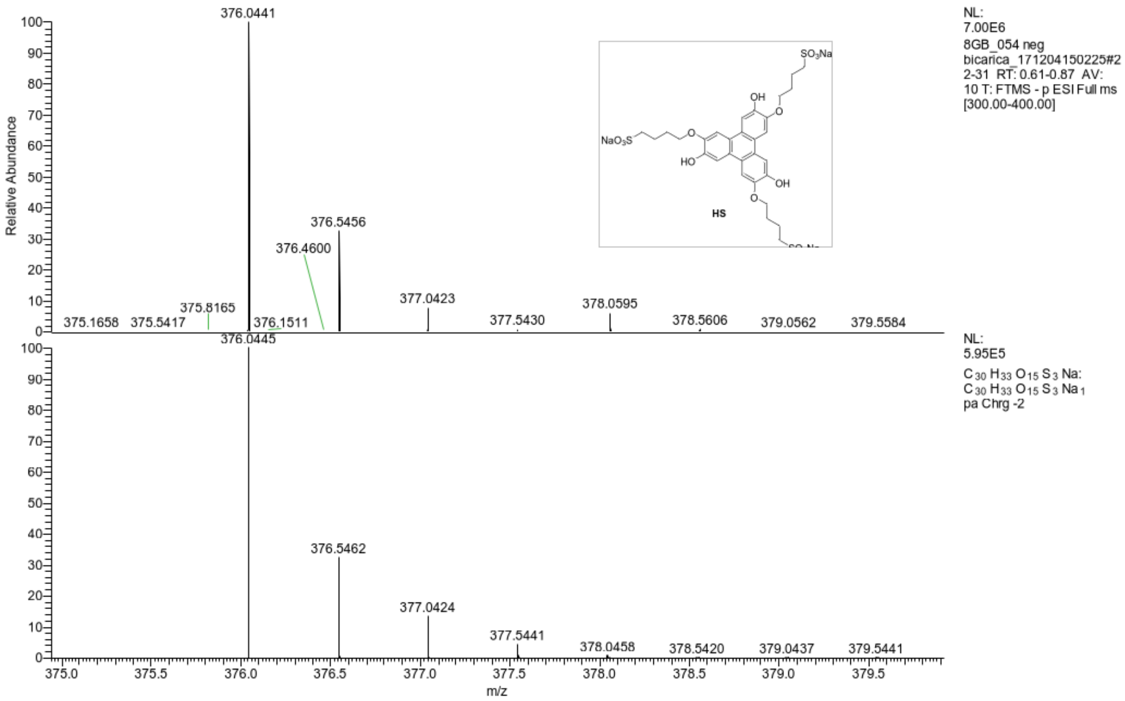

Figure S4: Mass spectra of HS - Part 1

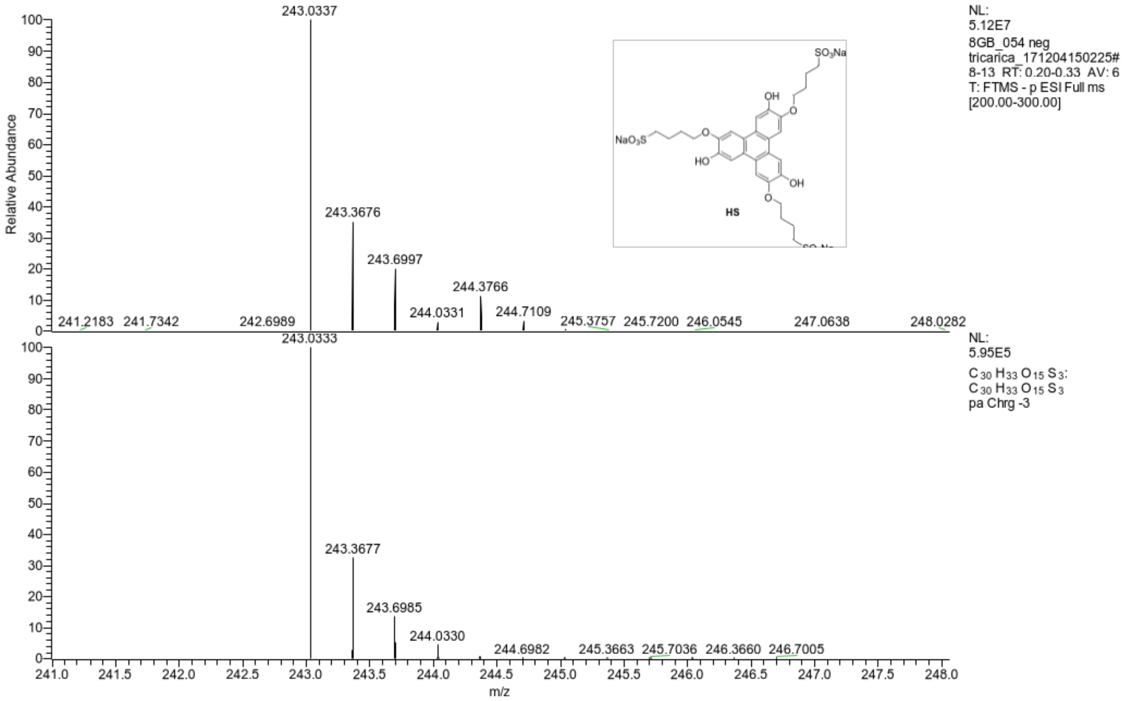

Figure S5: Mass spectra of HS - Part 2

S2.2 NMR

S2.2.1 BZS

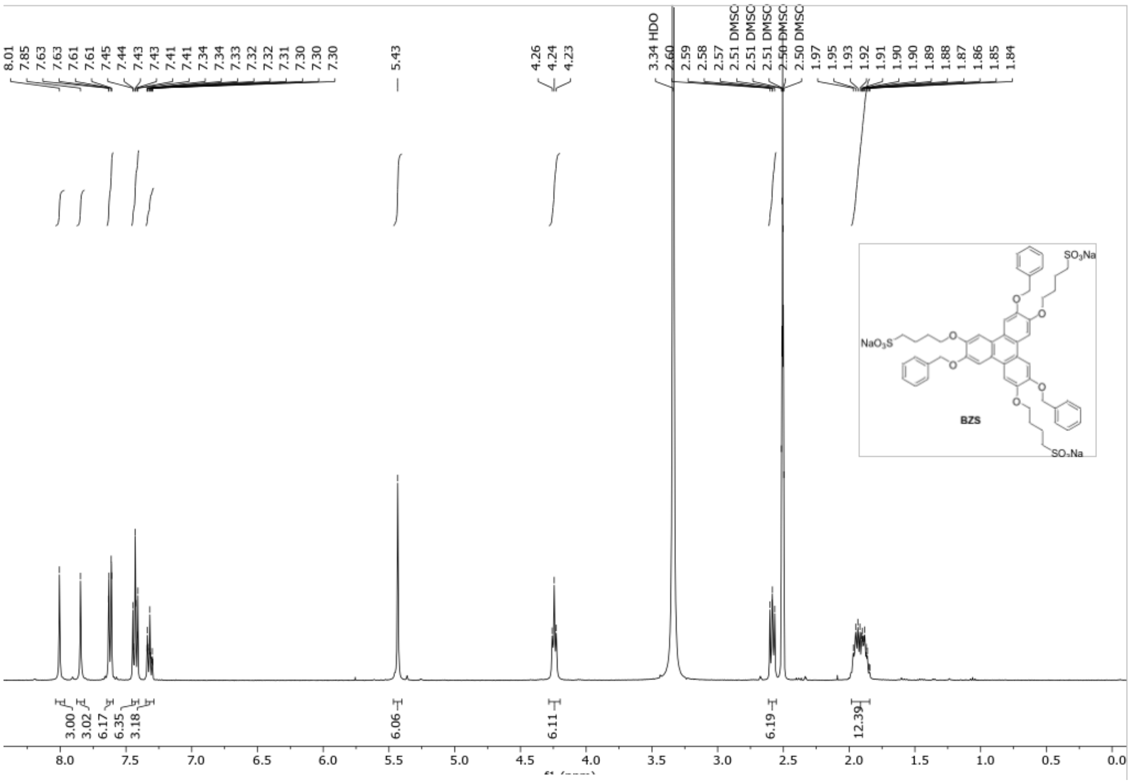

Figure S6: ^{1}H NMR spectrum of BZS

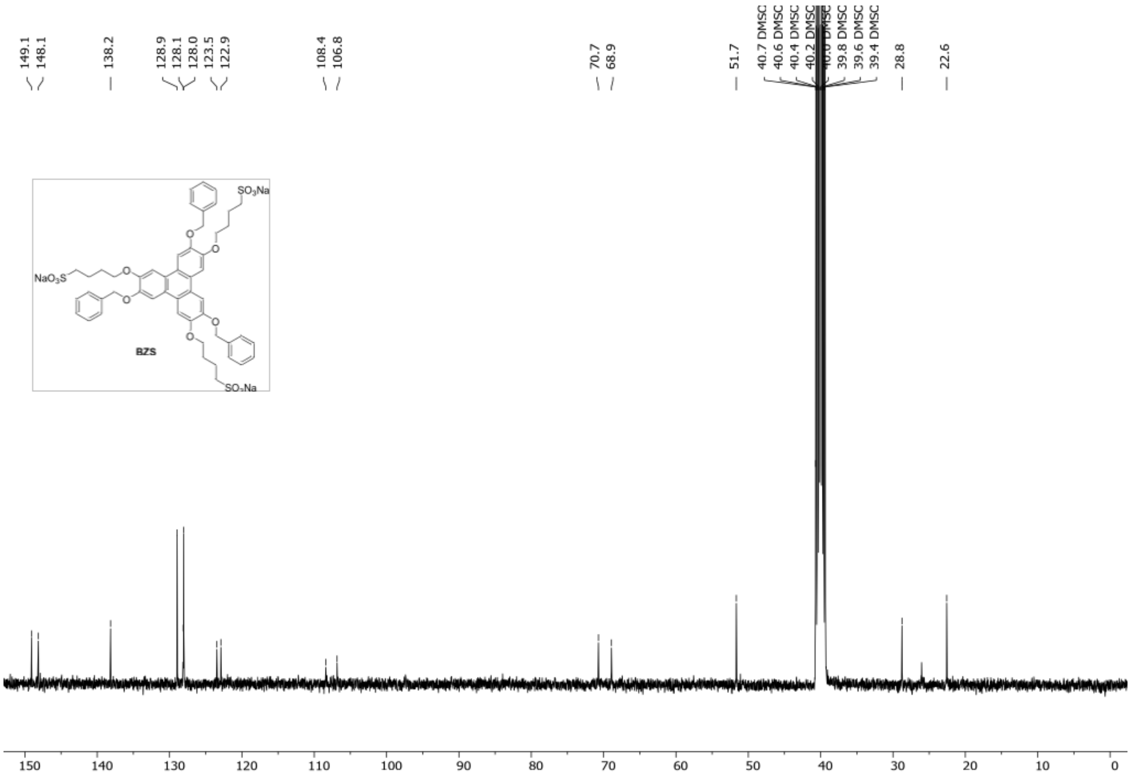

Figure S7: ^{1}H NMR spectra of BZS

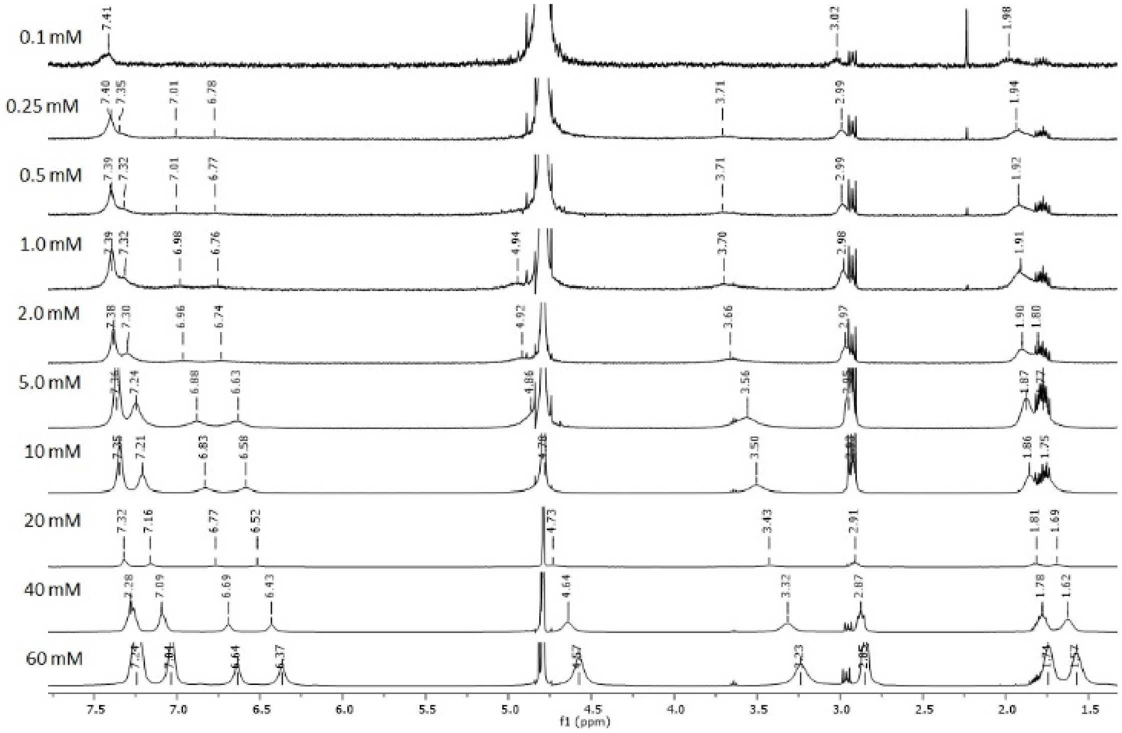

Figure S8: Summary of ^1H NMR spectra of BZS in D_2O as a function of the concentration.

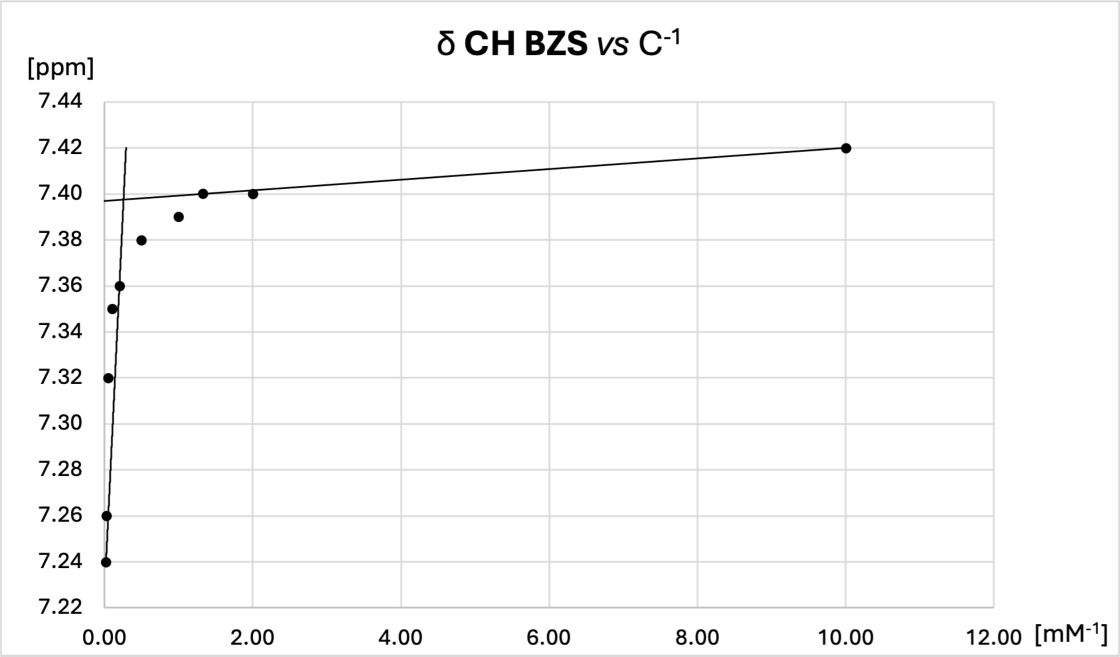

Figure S9: Plot of the aromatic CH chemical shift of BZS with respect to the inverse of the concentration. The two lines used to calculate the critical aggregation concentration are reported join at 3.9 mM.

S2.2.2 HS

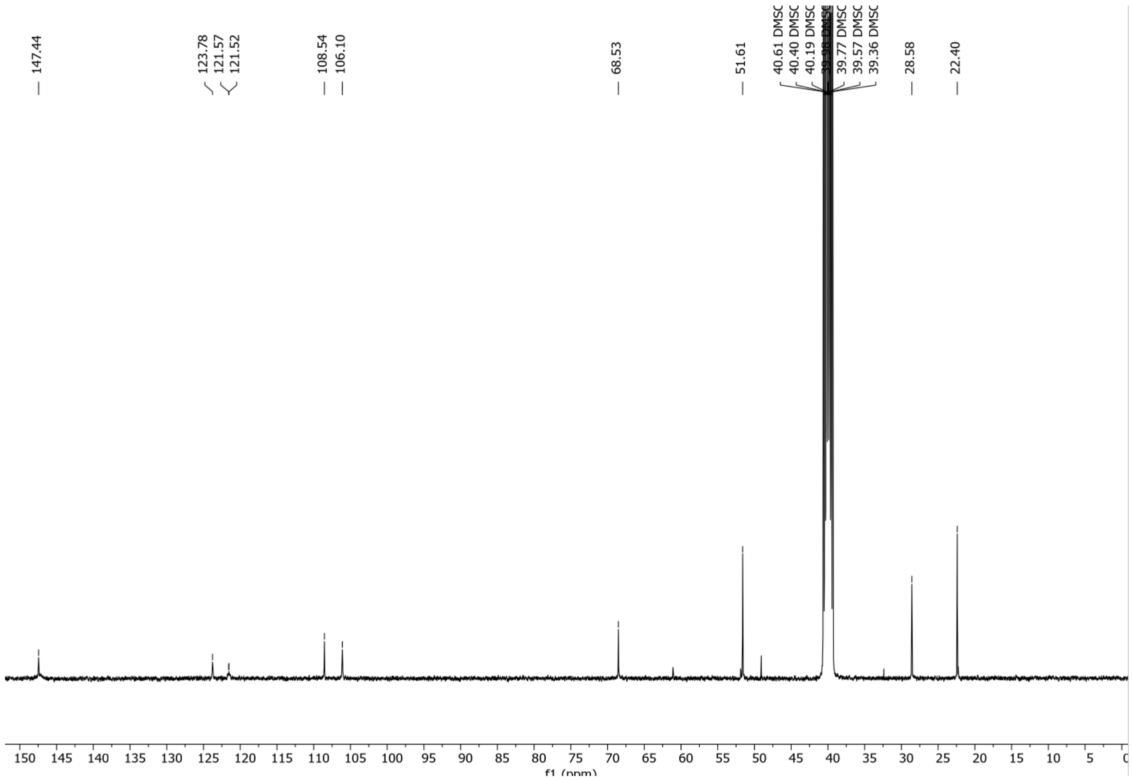

Figure S10: ^{13}C-NMR spectrum of HS in dsmo-d$_6$.

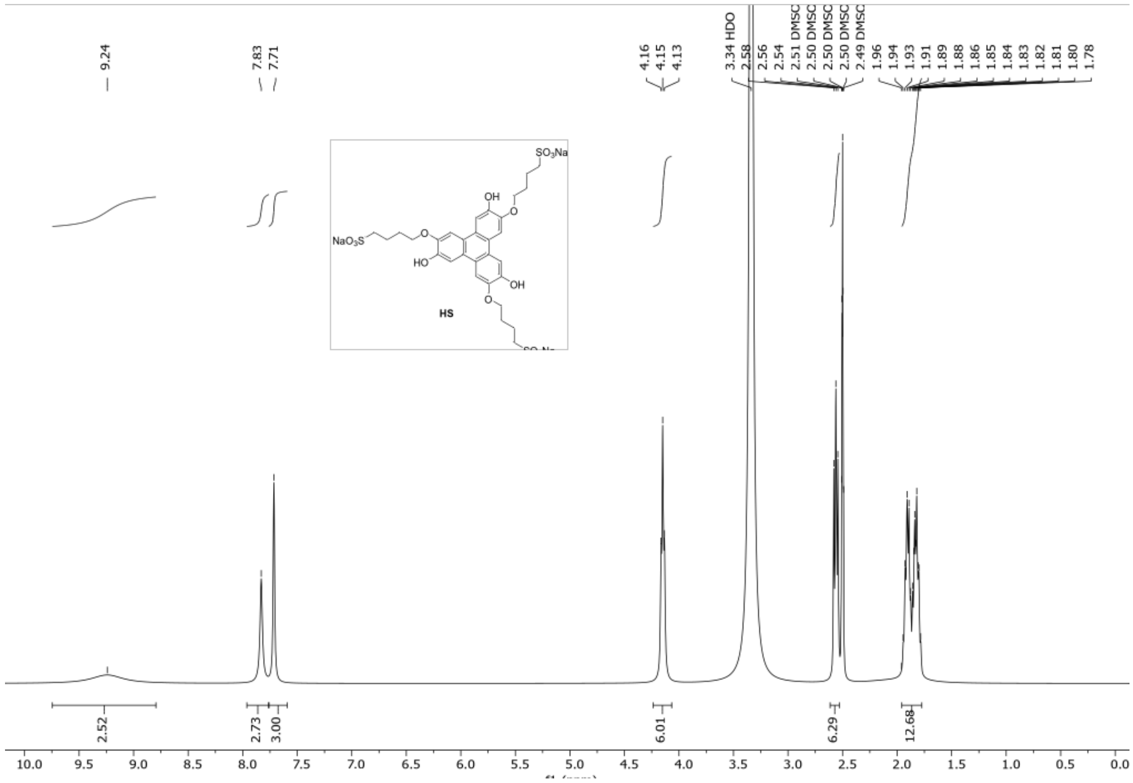

Figure S11: ^{1}H NMR spectra of HS

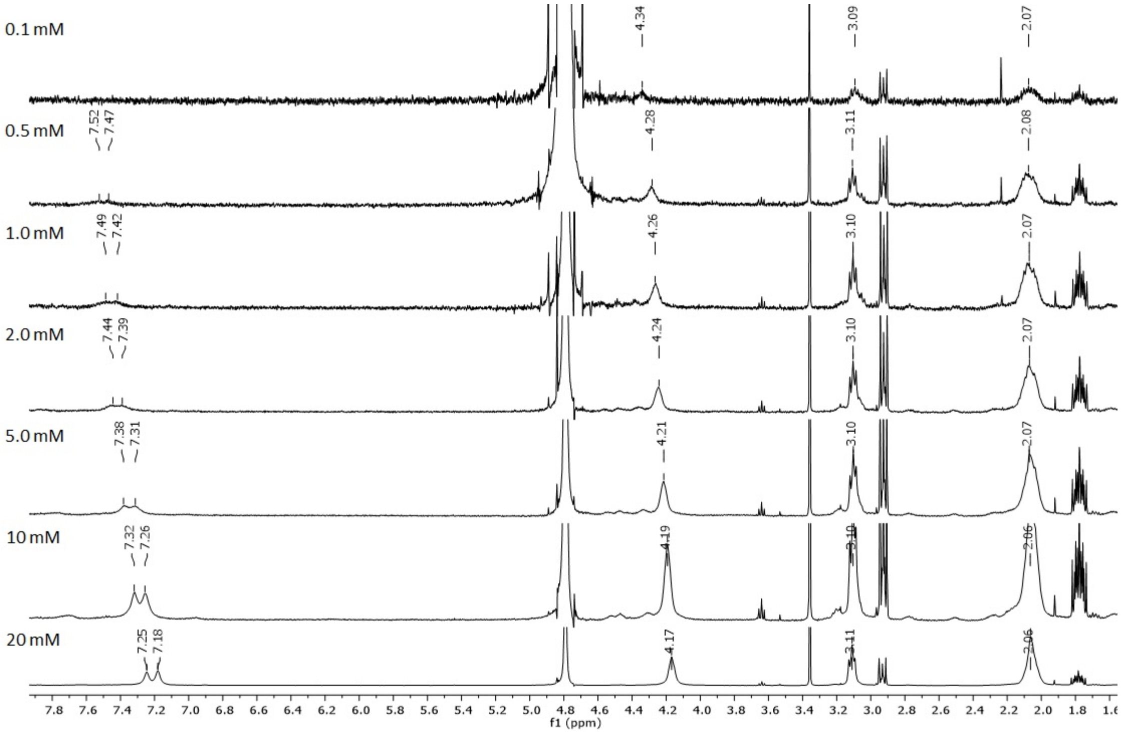

Figure S12: Summary of ^1H NMR spectra of HS in D_2O as a function of the concentration.

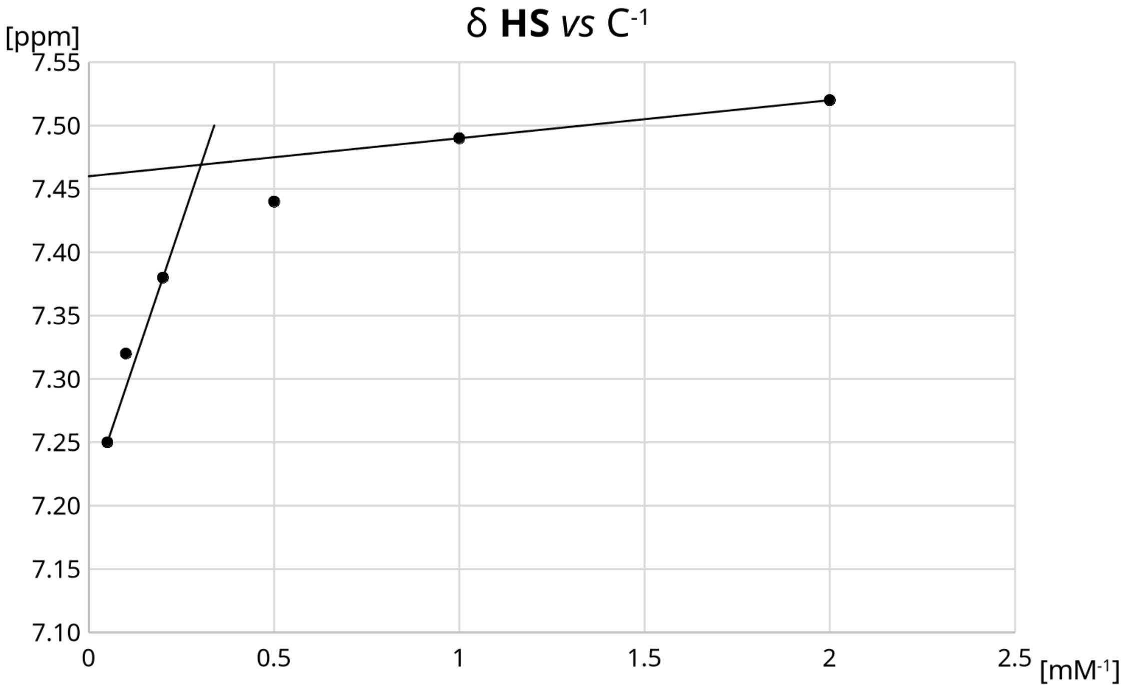

Figure S13: Plot of the aromatic CH chemical shift of HS with respect to the inverse of the concentration. The two lines used to calculate the critical aggregation concentration are reported join at 3.3 mM.

S2.3 DOSY NMR

S2.3.1 BZS

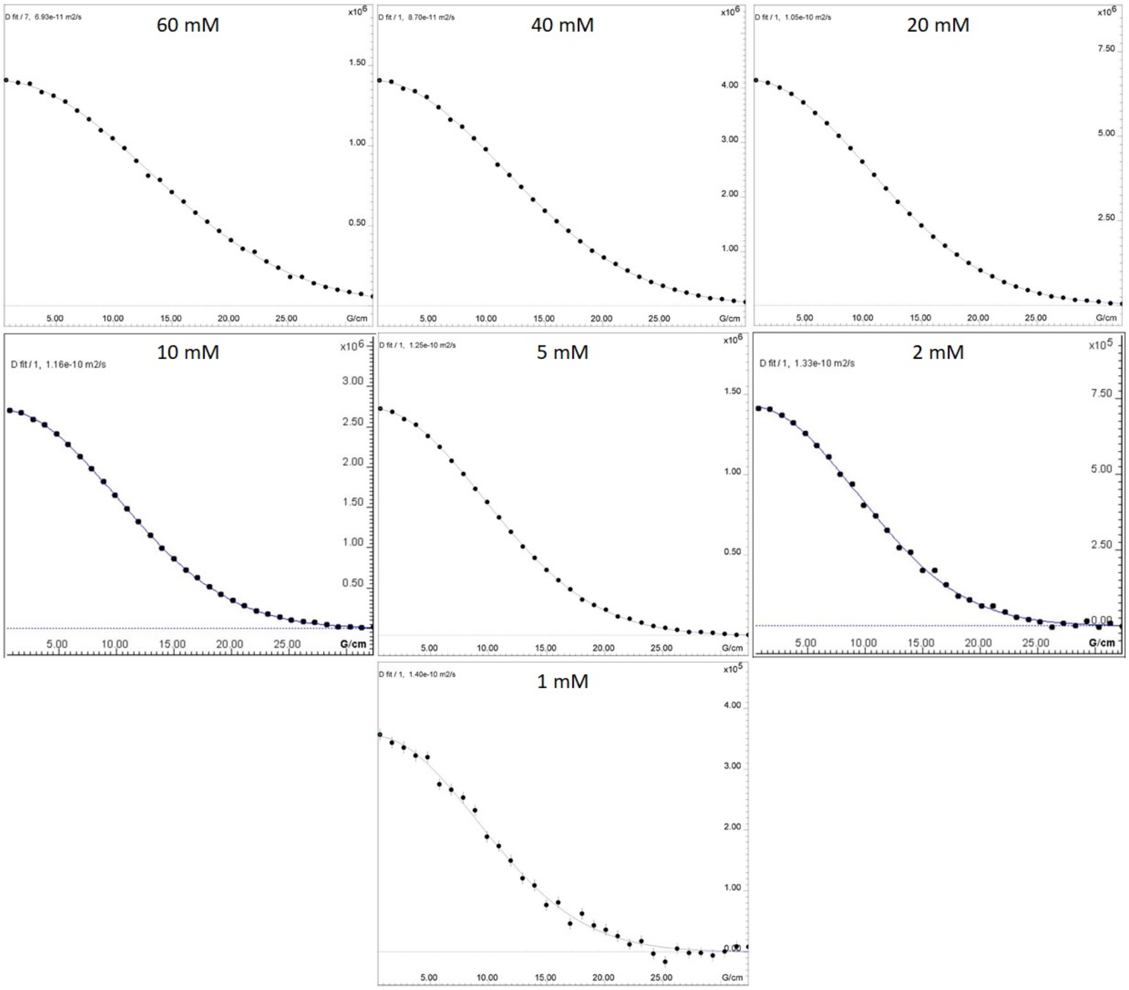

Figure S14: Pseudo2D-DOSY NMR mono-exponential decay of BZS benzyl signals in D_2O at different concentrations.

Table S1: Diffusion coefficients (D) of the two rounds of DOSY analysis of BZS in D_2O at different concentrations.

First Round	Second Round	C [mM]	Average D First [m^2s^{-1}]	Average D Second [m^2s^{-1}]	Average D [m^2s^{-1}]	StdDev D [m^2s^{-1}]	Error %
TGNGB008_60mM	TGNGB026_60mM	60	8.16E-11	6.88E-11	7.52E-11	9.06E-12	12.0
TGNGB008_40mM	TGNGB026_40mM	40	8.73E-11	8.68E-11	8.71E-11	2.99E-13	0.3
TGNGB008_20mM	TGNGB026_20mM	20	1.07E-10	1.06E-10	1.06E-10	1.22E-12	1.1
TGNGB008_10mM	TGNGB026_10mM_n2	10	1.19E-10	1.15E-10	1.17E-10	2.96E-12	2.5
TGNGB008_5mM	TGNGB026_5mM	5	1.27E-10	1.22E-10	1.25E-10	3.89E-12	3.1
TGNGB008_2mM	TGNGB026_2mM	2	1.37E-10	1.31E-10	1.34E-10	4.60E-12	3.4
TGNGB008_1mM	TGNGB026_1mM	1	1.42E-10	1.41E-10	1.42E-10	7.07E-13	0.5
TGNGB008_0,75mM_n2	TGNGB026_0,75mM	0.75	1.43E-10	1.45E-10	1.44E-10	1.65E-12	1.1
TGNGB008_0,5mM	TGNGB026_0,5mM	0.5	1.46E-10	1.53E-10	1.49E-10	5.30E-12	3.6
TGNGB008_0,1mM_n2	TGNGB026_0,1mM	0.1	2.19E-10	1.94E-10	2.07E-10	1.77E-11	8.6

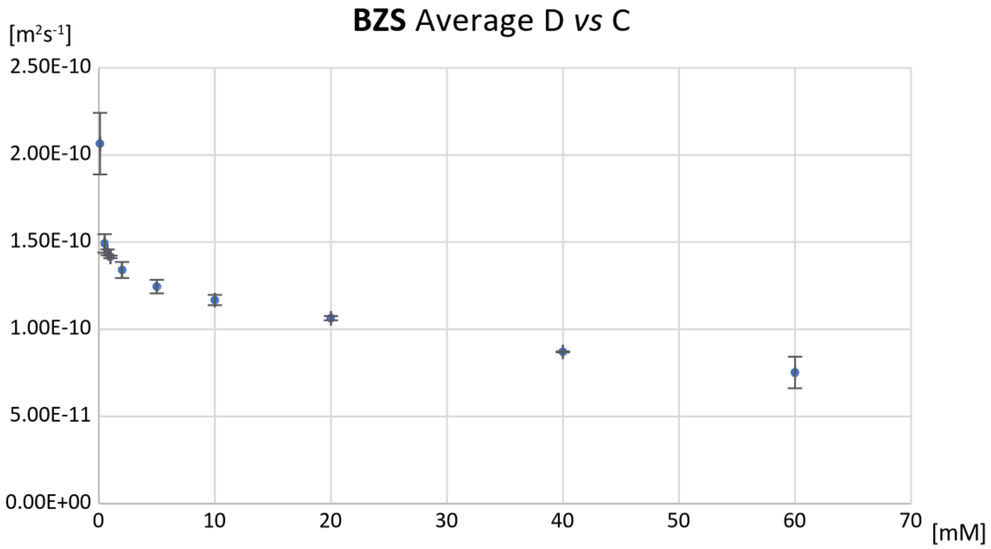

Figure S15: Diffusion coefficient of BZS as a function of concentration.

Table S2: Hydrodynamic radius of the two rounds of DOSY analysis of BZS in D_2O at different concentrations.

First Round	Second Round	C [mM]	Hydr. Radius First [m]	Hydr. Radius Second [m]	Average Hydr. Radius [m]	StdDev Hydr. Radius [m]	Error %
TGNGB008_60mM	TGNGB026_60mM	60	2.41E-09	2.86E-09	2.63E-09	3.17E-10	12.0
TGNGB008_40mM	TGNGB026_40mM	40	2.25E-09	2.26E-09	2.26E-09	7.74E-12	0.3
TGNGB008_20mM	TGNGB026_20mM	20	1.83E-09	1.86E-09	1.85E-09	2.12E-11	1.1
TGNGB008_10mM	TGNGB026_10mM_n2	10	1.65E-09	1.71E-09	1.68E-09	4.27E-11	2.5
TGNGB008_5mM	TGNGB026_5mM	5	1.54E-09	1.61E-09	1.58E-09	4.93E-11	3.1
TGNGB008_2mM	TGNGB026_2mM	2	1.43E-09	1.50E-09	1.47E-09	5.03E-11	3.4
TGNGB008_1mM	TGNGB026_1mM	1	1.38E-09	1.39E-09	1.39E-09	6.94E-12	0.5
TGNGB008_0,75mM_n2	TGNGB026_0,75mM	0.75	1.37E-09	1.35E-09	1.36E-09	1.56E-11	1.1
TGNGB008_0,5mM	TGNGB026_0,5mM	0.5	1.35E-09	1.28E-09	1.32E-09	4.68E-11	3.6
TGNGB008_0,1mM_n2	TGNGB026_0,1mM	0.1	8.97E-10	1.01E-09	9.55E-10	8.18E-11	8.6

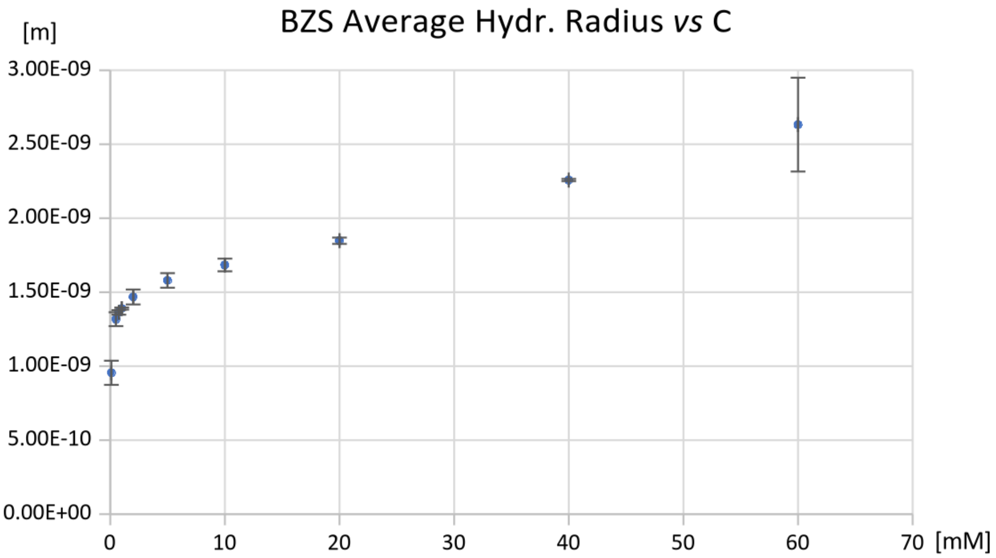

Figure S16: Hydrodynamic radius of BZS as a function of concentration.

S2.3.2 HS

Table S3: Diffusion coefficients (D) of the two rounds of DOSY analysis of HS in D_2O at different concentrations.

First Round	Second Round	C [mM]	Average D First [m^2s^{-1}]	Average D Second [m^2s^{-1}]	Average D [m^2s^{-1}]	StdDev D [m^2s^{-1}]	Error %
TGNGB025_20mM	TGNGB043_20mM_n2	20	1.70E-10	2.16E-10	1.93E-10	3.31E-11	17.1
TGNGB025_10mM	TGNGB043_10mM	10	1.70E-10	1.69E-10	1.69E-10	8.49E-13	0.5
TGNGB025_5mM	TGNGB043_5mM	5	1.74E-10	1.73E-10	1.73E-10	9.43E-13	0.5
TGNGB025_2mM_n2	TGNGB043_2mM	2	1.73E-10	1.85E-10	1.79E-10	8.49E-12	4.7
TGNGB025_1mM_n2	TGNGB043_1mM	1	1.90E-10	2.22E-10	2.06E-10	2.26E-11	11.0
TGNGB025_0,5mM	TGNGB043_0,5mM	0.5	2.13E-10	2.21E-10	2.17E-10	5.66E-12	2.6
TGNGB025_0,1mM	TGNGB043_0,1mM	0.1	3.11E-10	3.26E-10	3.19E-10	1.06E-11	3.3

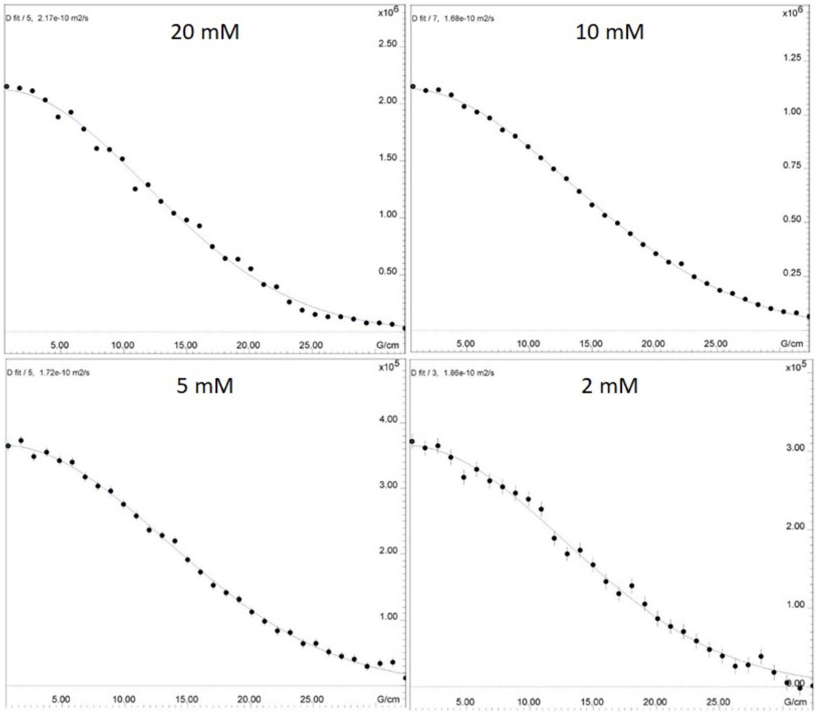

Figure S17: Pseudo2D-DOSY NMR mono-exponential decay of HS triphenylenic signals in D_2O at different concentrations.

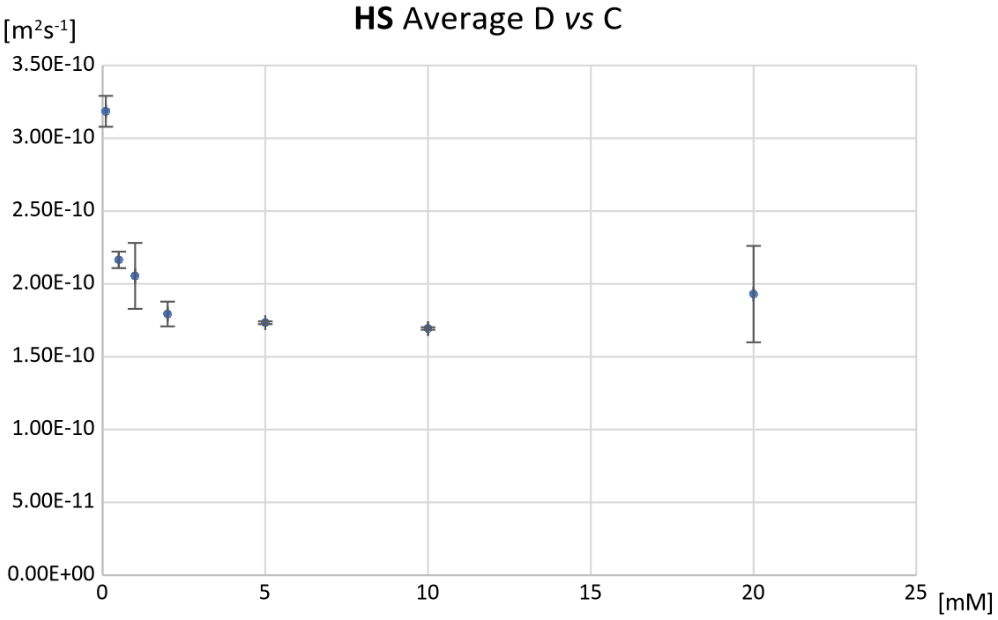

Figure S18: Diffusion coefficient of HS as a function of concentration.

Table S4: Hydrodynamic radius of the two rounds of DOSY analysis of HS in D_2O at different concentrations.

First Round	Second Round	C [mM]	Hydr. Radius First [m]	Hydr. Radius Second [m]	Average Hydr. Radius [m]	StdDev Hydr. Radius [m]	Error %
TGNGB025_20mM	TGNGB043_20mM_n2	20	1,16E-09	9,08E-10	1,03E-09	1,77E-10	17,1
TGNGB025_10mM	TGNGB043_10mM	10	1,16E-09	1,16E-09	1,16E-09	5,81E-12	0,5
TGNGB025_5mM	TGNGB043_5mM	5	1,13E-09	1,14E-09	1,13E-09	6,17E-12	0,5
TGNGB025_2mM_n2	TGNGB043_2mM	2	1,13E-09	1,06E-09	1,10E-09	5,19E-11	4,7
TGNGB025_1mM_n2	TGNGB043_1mM	1	1,04E-09	8,87E-10	9,62E-10	1,06E-10	11,0
TGNGB025_0,5mM	TGNGB043_0,5mM	0,5	9,25E-10	8,91E-10	9,08E-10	2,37E-11	2,6
TGNGB025_0,1mM	TGNGB043_0,1mM	0,1	6,32E-10	6,03E-10	6,17E-10	2,06E-11	3,3

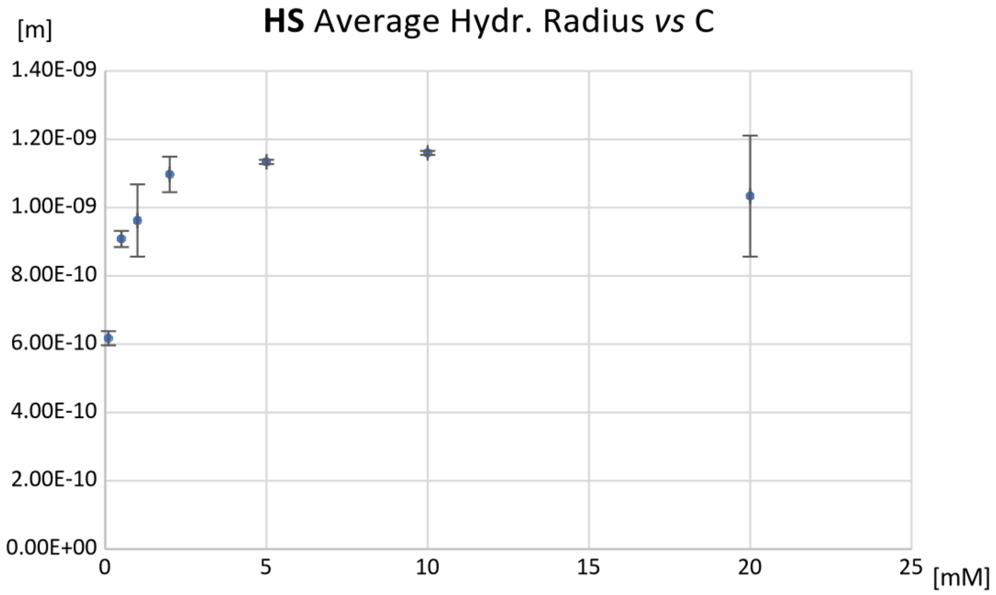

Figure S19: Hydrodynamic radius of HS as a function of concentration.

S2.4 UV-vis and fluorescence

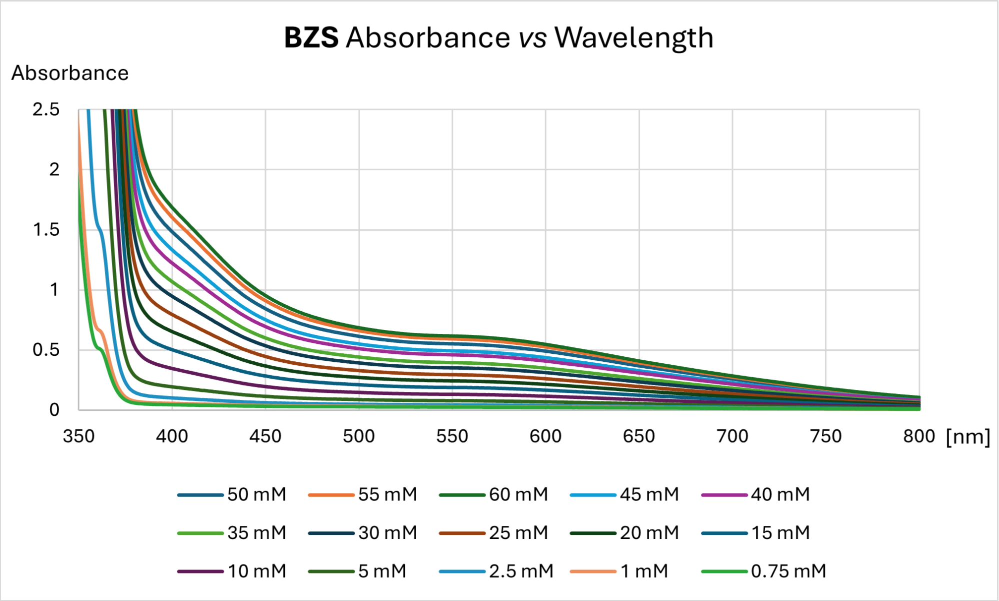

Figure S20: UV-Vis spectra of BZS in water at different concentrations at 297 K.

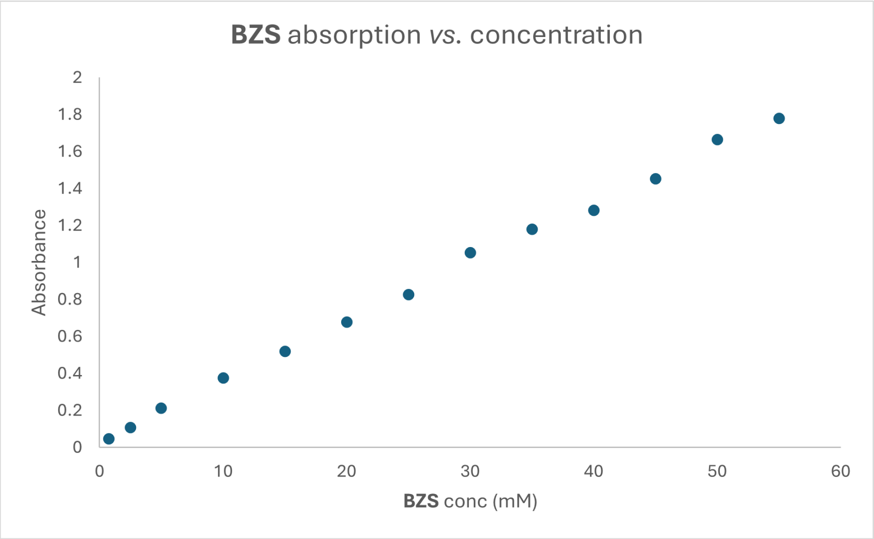

Figure S21: Plot of Absorbance vs. concentration for BZS in water.

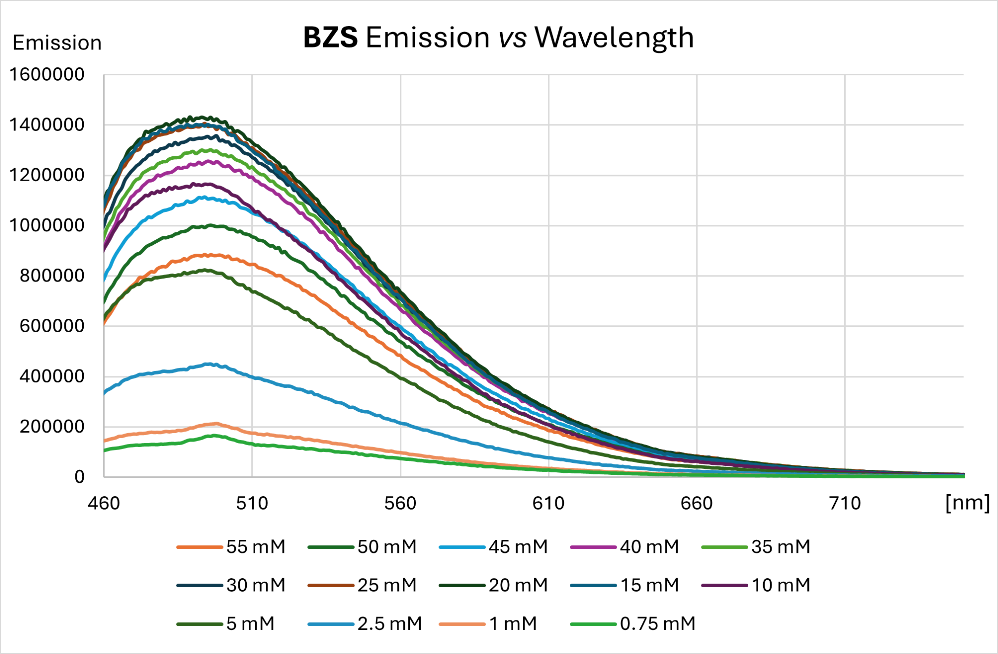

Figure S22: Emission spectra of BZS in water at different concentrations at 297 K. Excitation wavelength 425 nm.

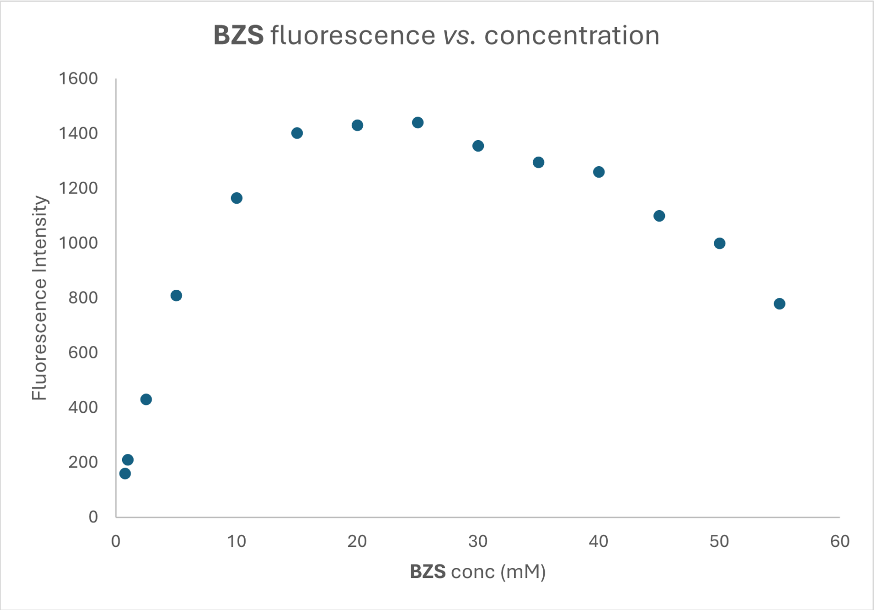

Figure S23: Plot of Fluorescence intensity vs. concentration for BZS in water.

S2.5 Small-angle scattering

S2.5.1 Model fitting

Porod exponent analysis and curve fitting The SANS I(Q) data was analysed with SasView software.[S1] A full description of the data was obtained combining a core-shell sphere model for the form factor (CSS) to describe the dimension of the primary particles (called micelles) and the Hayter-Penfold mean spherical approximation (Hmsa) model for the structure factor to describe the interaction between the charged particles. A power law has been added to fit the scattering contribution coming from larger aggregates by using the Porod exponent analysis.[S2] The results are summarized in Table S5.

Table S5: Fit results for the BZS 25 mM solution in D2O, using the CSSxHmsa model.

**Radius of core [nm]**	**Thickness of shell [nm]**	**Polidispersity of radius**	**Polydispersity of thickness**
1.13±0.01	0.77±0.09	0.01±0.03	0.5±0.1
**SLD core /10^6 [Å^{-2}]**	**SLD shell /10^6 [Å^{-2}]**	**Radius effective [nm]**	**Charge** [e]
2.2±0.2	5.97±0.04	1.8±0.1	12.4±0.9
**Porod exponent**		χ^2	
2.8±0.2		1.45	

In the low-Q region the scattering curve exhibits a power law behavior proportional to Q^{-a}, with a = 2.8±0.2, suggesting a mass fractal behavior. This suggests the presence of mass-fractal aggregation of the primary particles (composed of approx. 4 units each, with a total charge of ca 12 e, see below) aggregated in a core–shell fashion (eventually/probably stacked one over each other). These particles are composed of a hydrophilic core of radius of (1.128±0.007) nm and a hydrophobic shell of thickness of (0.77±0.09) nm. The mean value of the effective radius of these free nano-micelles is (1.8±0.1) nm. Furthermore, from our model, the micelles carry a charge of (12.4±0.9) e.

Kratky Plot analysis The incoherent background was subtracted before doing a Kratky plot (Figure S25). It shows the presence of two peaks (at Q of 0.1258 and 0.2242 Å^{-1}) indicating a compact spherical structure of the primary particles, in agreement with results from simulations. The existence of two peaks could be explained by two similar but distinct

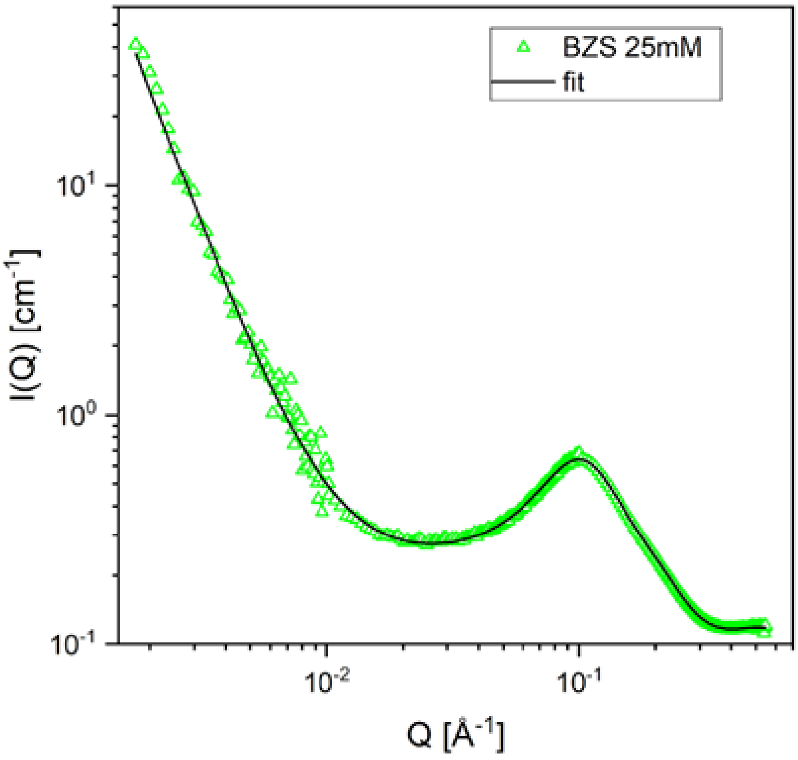

Figure S24: SANS data (green triangles) and the best fit (black line) for the sample BZS 0.25 mM.

structures. From the peak positions, we obtain the pseudo-Guinier radii $R_{\text{pg}} = \frac{\sqrt{3}}{Q_{\text{max}}}$,[S3] resulting in 1.38 nm and 0.77 nm. These two length values are in good agreement with the core radius and shell thickness calculated from the fitting described above, i.e. (1.128±0.007) nm and (0.77±0.09) nm, respectively (Table S5).

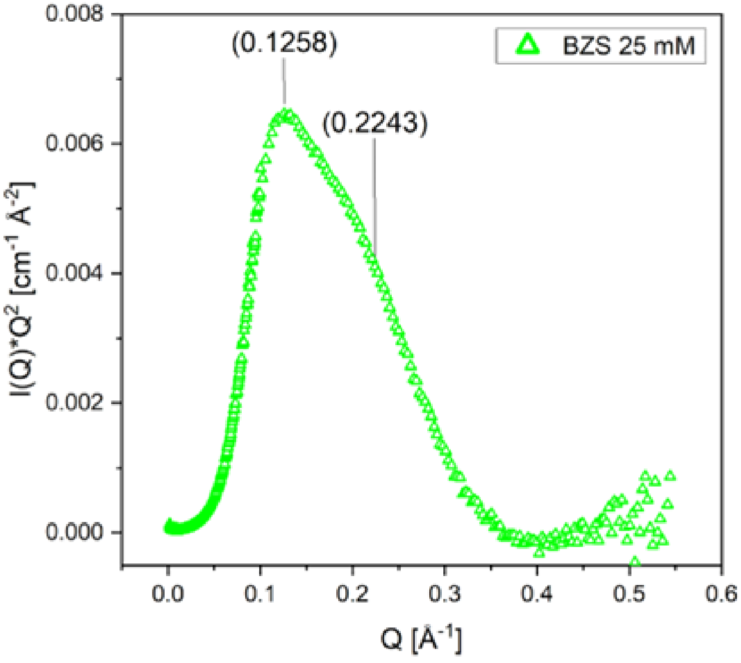

Figure S25: Kratky plot of the BZS 0.25 mM sample.

Stacked disk form factor fitting We also include an unsuccessful fit using the stacked disk model, shown in Figure S26. Details about the employed model can be found in the literature.[S4–S7] Results from the "best" fit (obtained after several trials) are summarized in Table S6. Using this model, it was not possible to fit the SANS data satisfactorily.

Table S6: Fit results for the BZS 25 mM solution in D_2O, using the stacked disk model.

**Radius [nm]**	**Thickness of layer [nm]**	**Thickness of core [nm]**	**SLD core /10^6 [Å^{-2}]**
1.0±0.5	0.2±0.2	5±4	1.5±0.9
**Polidispersity of radius**	**Polydispersity of thickness**	**Charge [e]**	**SLD layer /10^6 [Å^{-2}]**
0.2±0.2	0.5±0.5	15±5	2±1
χ^2			
20			

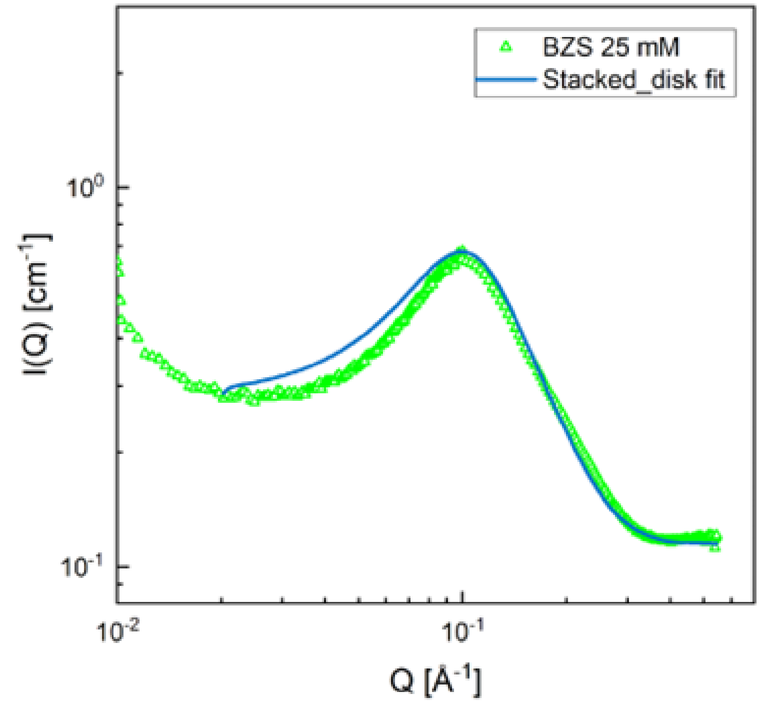

Figure S26: SANS data (green triangles) and the fit (black line) using the stacked disk model for the sample BZS 0.25 mM.

S2.5.2 SAXS additional plots

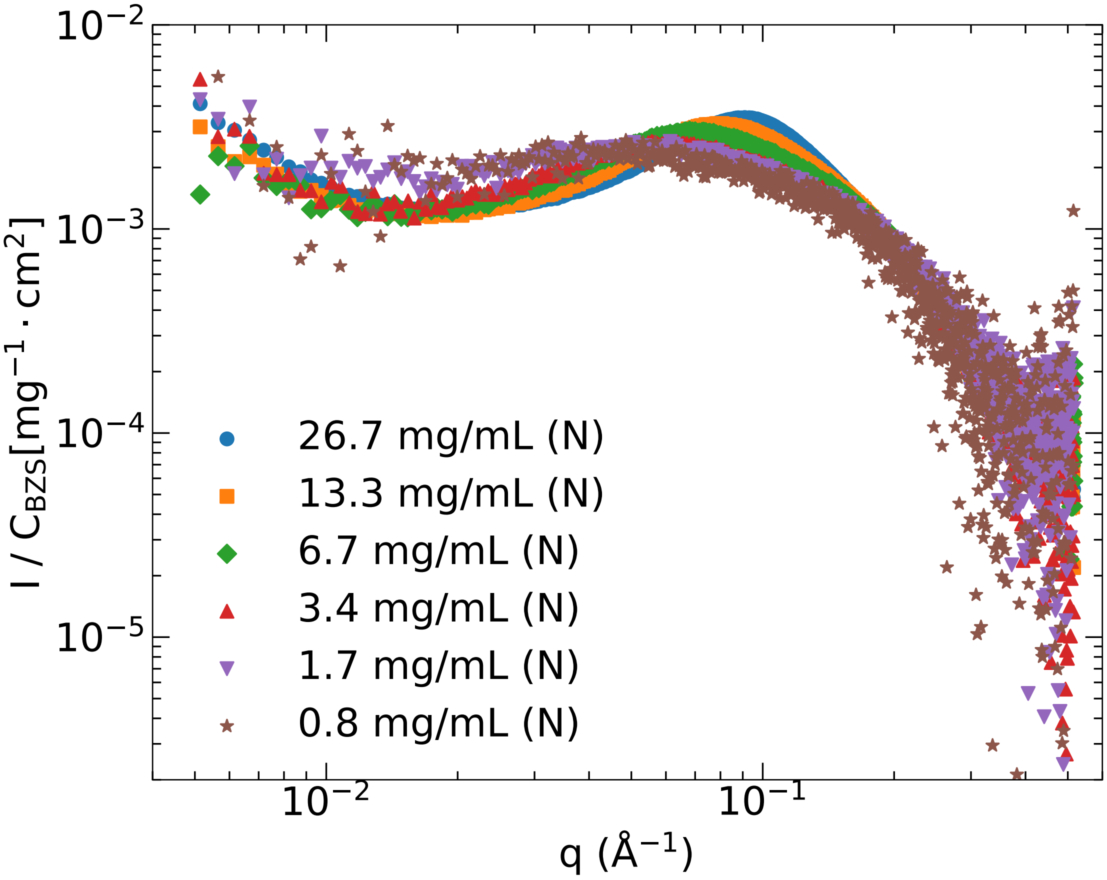

Figure S27: SAXS curves for BZS solutions, normalized by dividing the intensity by solution concentration

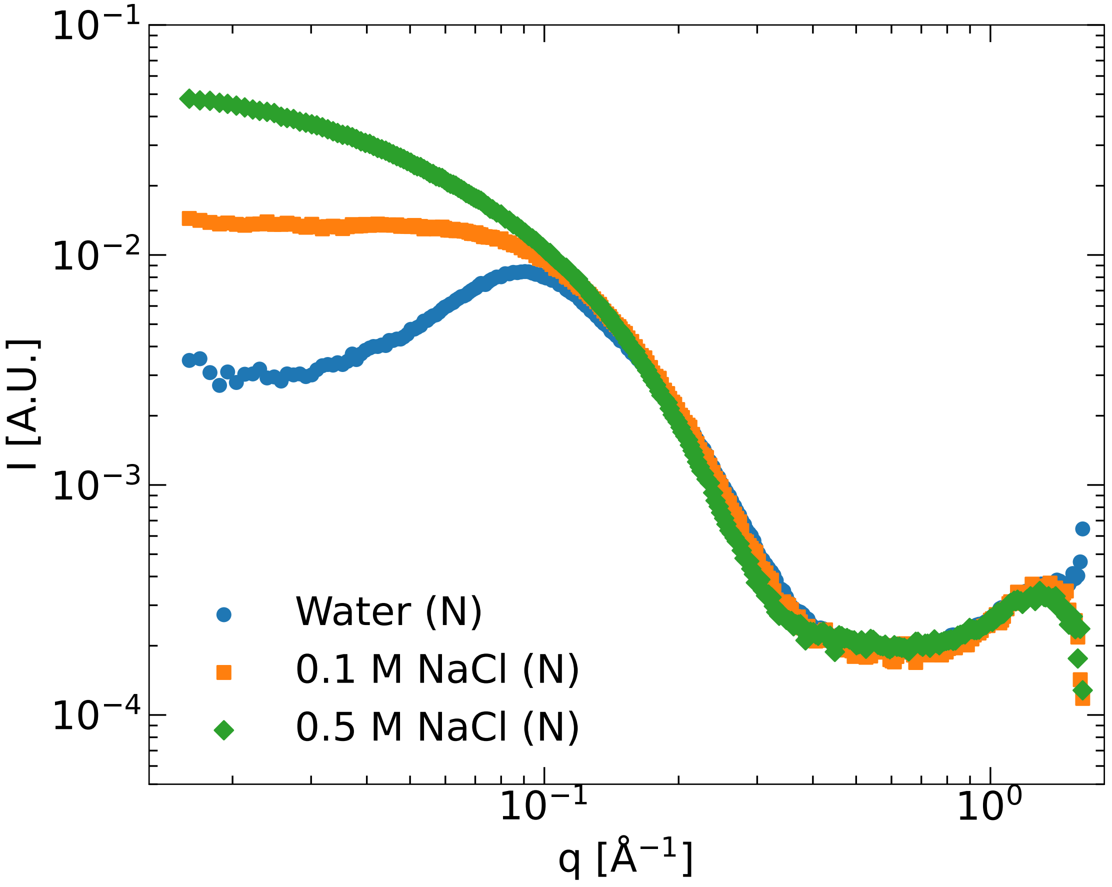

Figure S28: SAXS curves for BZS solutions, normalized to have the same relative contrast between BZS and solvent

S3 Simulation results

S3.1 Aggregation analysis for coarse grained simulations

Aggregate analysis Using hierarchical clustering, aggregates were automatically detected from the coarse-grained trajectories using Python. We used MDAnalysis[S8,S9] to handle the trajectories and calculate the distances, and SciPy[S10] to clustering. The tool is available as a utility for HyMD[S11,S12]. A total of 400 frames were analyzed, with the distances between molecules being computed based on distances of one of the central virtual sites, and using the unweighted pair group method with arithmetic mean (UPGMA) for linking. We used a clustering criterion of 20 Å, which showed a good balance between the number of clusters and the spatial distribution of molecules belonging to the aggregate. Based on the aggregate size, we also obtained radial distribution functions to determine the density of different groups with respect to the micelle core.

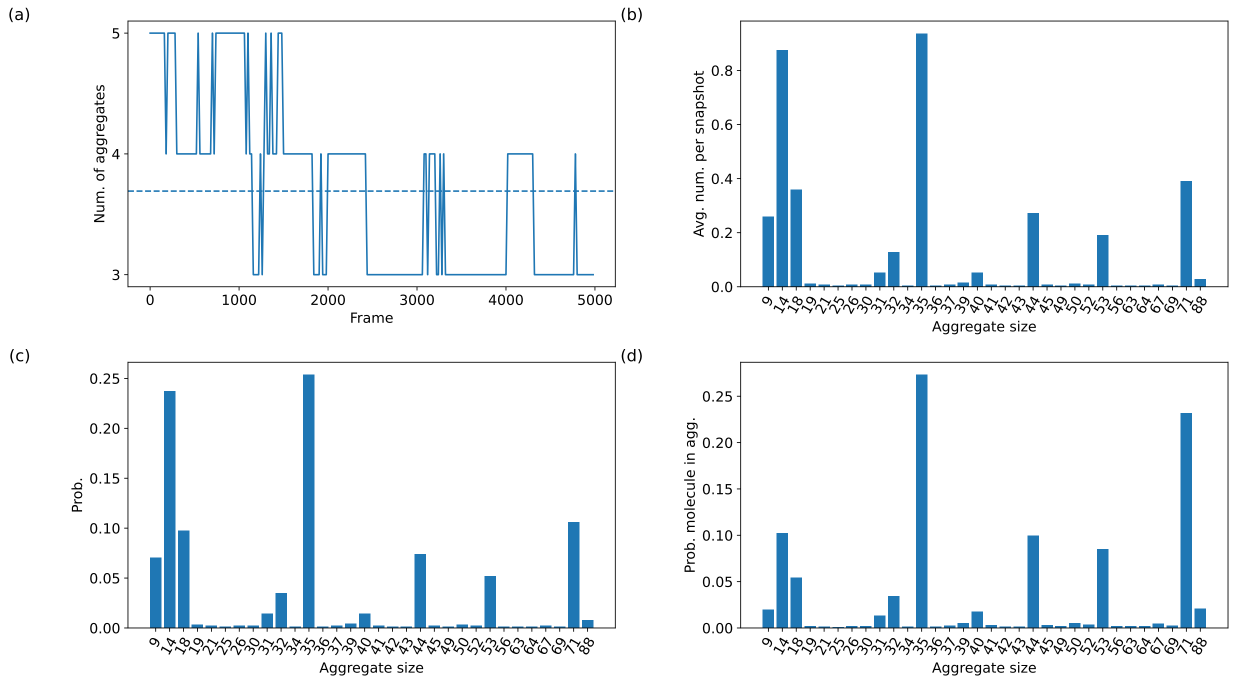

Figure S29: Aggregate analysis from the unbiased Martini 3 simulations trajectories in water: (a) number of detected aggregates for trajectory frames; (b) average number of aggregates with given number of BZS molecules per snapshot; (c) probability of finding aggregate of given size in each snapshot; (d) probability of having a BZS molecule belonging to an aggregate of a given size.

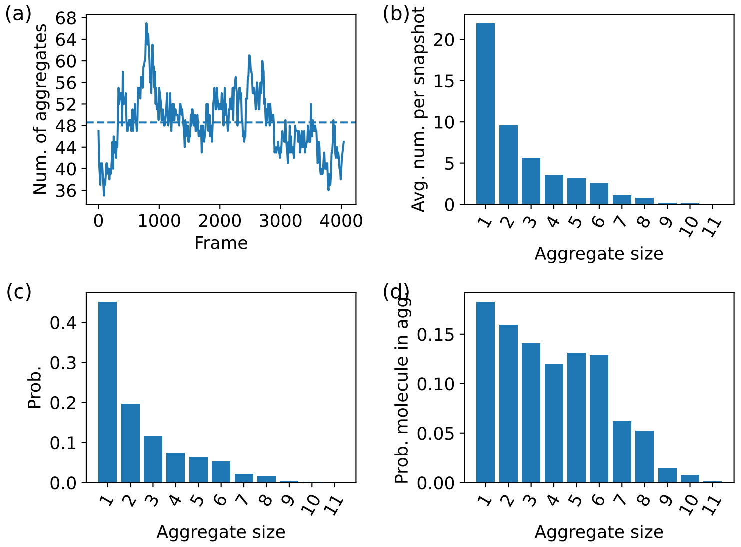

Figure S30: Aggregate analysis from the Metainference simulations trajectories in water: (a) number of detected aggregates for trajectory frames; (b) average number of aggregates with given number of BZS molecules per snapshot; (c) probability of finding aggregate of given size in each snapshot; (d) probability of having a BZS molecule belonging to an aggregate of a given size.

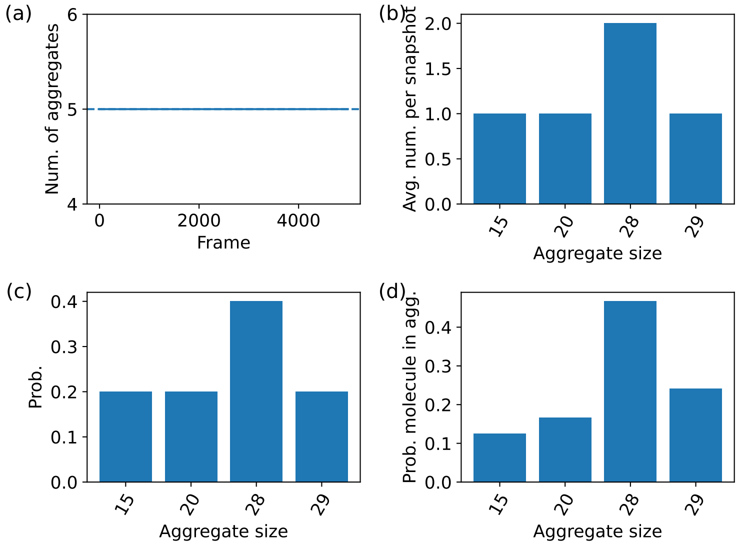

Figure S31: Aggregate analysis from the Metainference simulations trajectories in 0.1 M NaCl solution: (a) number of detected aggregates for trajectory frames; (b) average number of aggregates with given number of BZS molecules per snapshot; (c) probability of finding aggregate of given size in each snapshot; (d) probability of having a BZS molecule belonging to an aggregate of a given size.

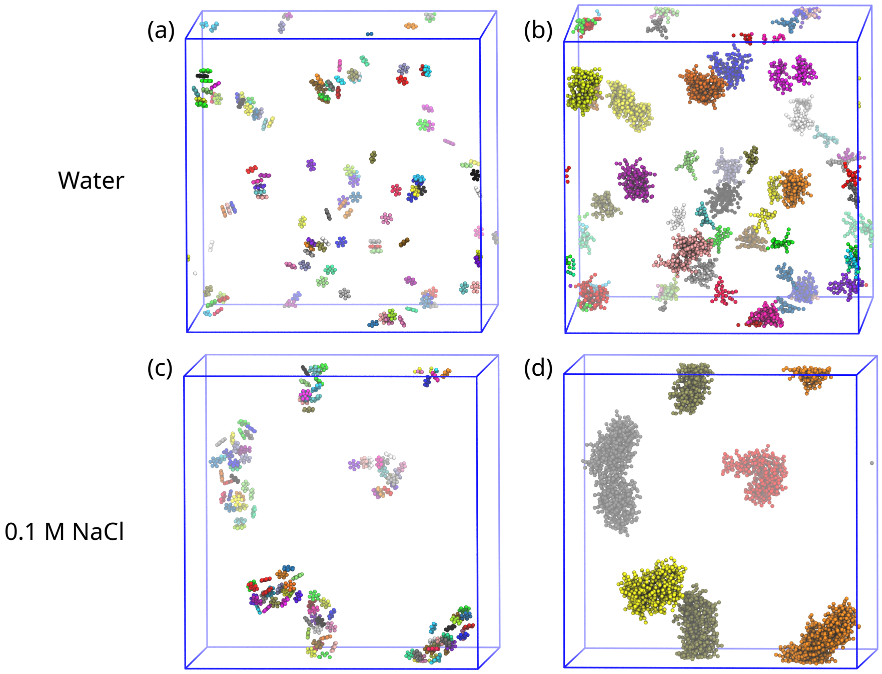

Figure S32: Example snapshots from the coarse-grained Metainference simulations. (a) and (c) the beads representing the polycyclic core, colored by molecule; and (b) and (d) all BZS beads colored by aggregate. The snapshots in (a) and (b) are from the water simulations, while the snapshots in (c) and (d) are for the simulations in 0.1 M NaCl solution. Water and ion beads were removed from the visualization for clarity.

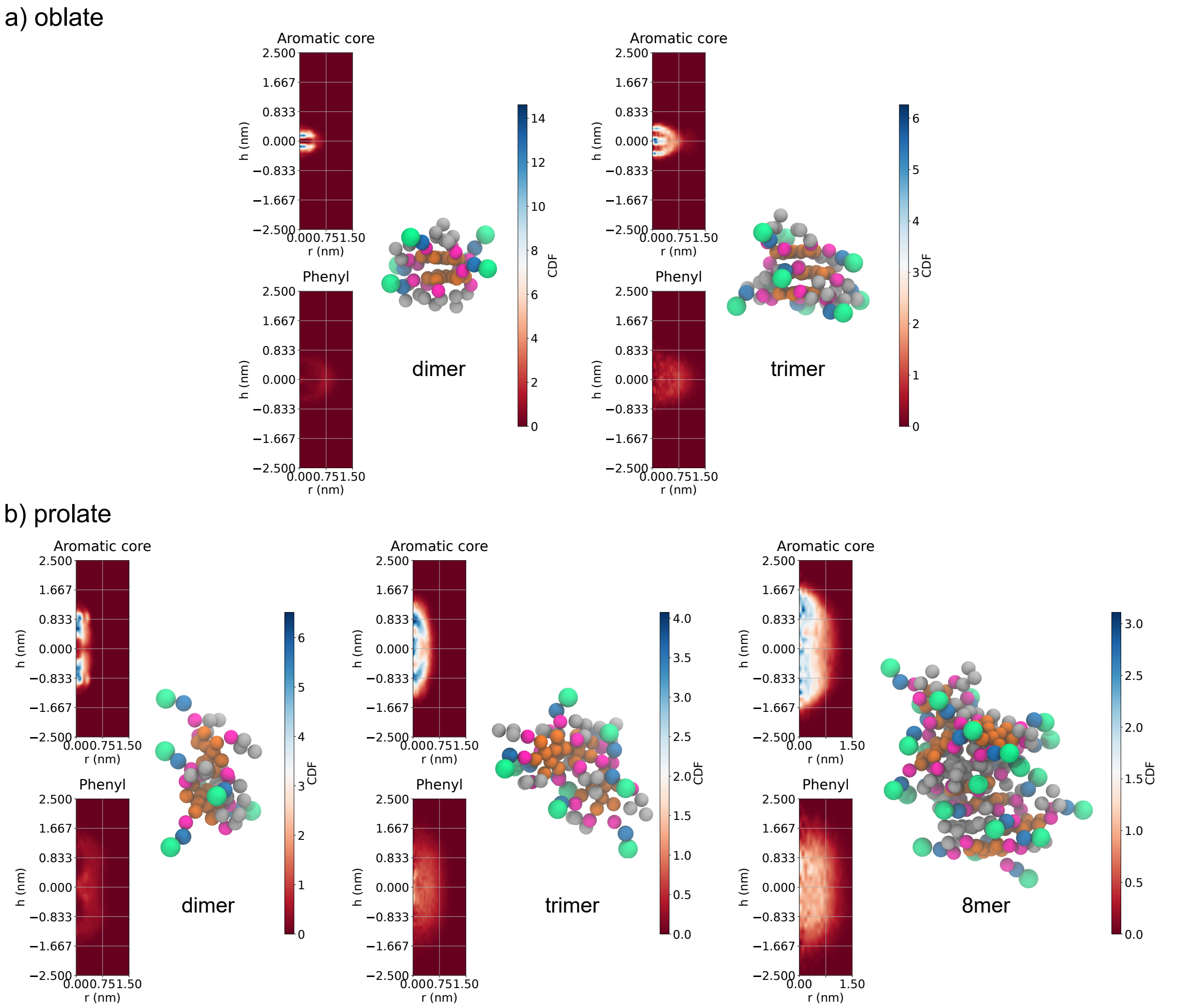

Figure S33: CDFs and example snapshot for selected sizes from the Metainference BZS simulation in pure water. a) oblate aggregates for the dimer and trimer. b) prolate aggregates for the dimer, trimer and *8mer*. The axis of symmetry are aligned along the vertical. The bead color of the example configurations follow the bead types of Figure 3b of the main text.

S3.2 Conformational differences in water and NaCl solution

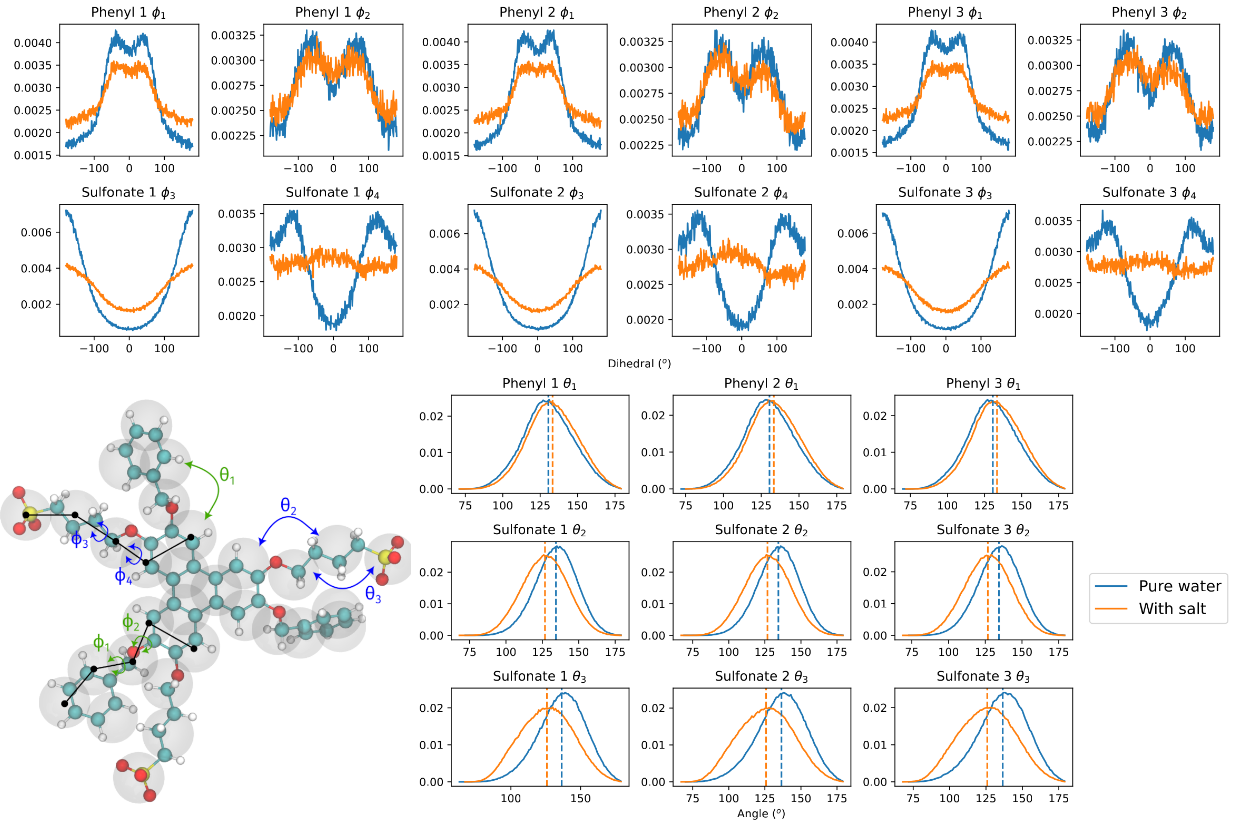

Figure S34: Selected angles and dihedral angles distribution sampled in the Metainference simulations in water and 0.1 M NaCl solution. Labels for the angles are given in the atomic structure and represent angles of the coarse-grained structure (displayed in semi-transparent grey). The labels are separated for the phenyl and sulfonate groups. Vertical dashed lines for the angles represent the average.

S3.3 BZS simulation with salt

To be straightforwardly compared with coarse-grained metainference simulations, we performed a 100 ns atomistic simulation of BZS 25mM with OPLS-AA/TIP4P and 0.1 M NaCl using the same simulation parameters. The results are reported in Fig. S35 below. It turns out that the inclusion of salt in the simulation consistently drives the decrease of the available solvent accessible surface area of BZS (Fig. S35-a), a sign of tighter packaging, along with a remarkable increase in the aggregation size of BZS-BZS, going from 4 in the case of pure water to 7 after salt addition (Fig. S35-b). Moreover, the BZS-BZS pair radial

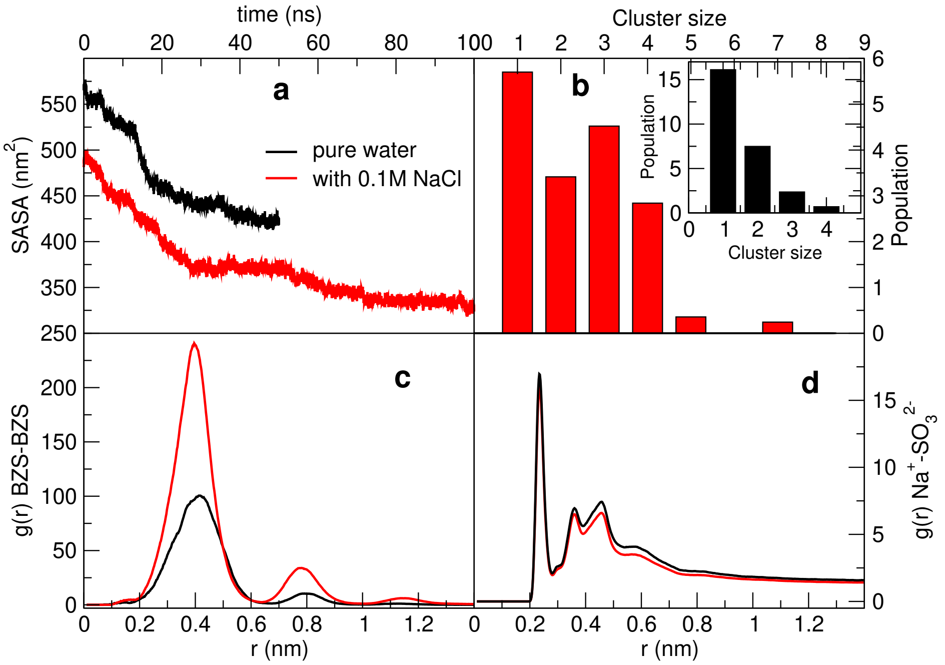

Figure S35: Order parameters for all-atom simulations of BZS self-assembly process in pure water (black) and with 0.1M NaCl (red). (a) The time-based total solvent accessible surface area of BZS entities; (b) The cluster size distribution of BZS aggregate in simulation with salt and in pure water (in the inset); (c) The BZS-BZS center of mass pair radial distribution function; (d) Na^+-SO_3^{2-} pair radial distribution function.

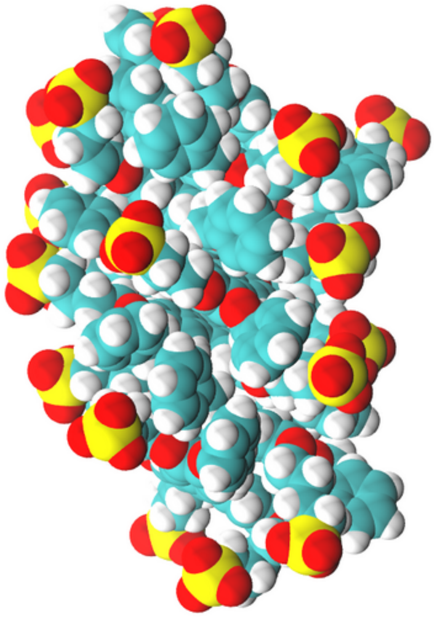

Figure S36: Representative snapshot and close-up view of the largest 7mer-BZS identified in all-atom simulation of BZS in water with 0.1M NaCl.

distribution function shows a more intense first peak compared to the pure water case (Fig. S35-c), pointing to more interacting BZS units in the first coordination shell. It should be noted that Na^+ cations show favorable contact in the first coordination shell around 0.25 nm with the sulfonate anions SO_3^{2-} witnessed by the sharp but intense peak in Na^+-SO_3^{2-} rdf (Fig. S35-d). Strikingly, as shown in Fig. S36, the stacked aggregate is evidently more tightly packed and distorted compared to the pure water case, which is well correlated with the morphology change observed in coarse-grained metainference simulations. Altogether, the results of the atomistic simulation with salt agree strongly with those of coarse-grained metainference counterparts.

S3.4 Atomistic Potential of Mean Force

The simulation performed on a 25mM solution made of 41 moieties of BZS randomly distributed in a cubic box prevalently lead to a 4mer-stacked BZS assemblies, the aggregation propensity of which was assessed by computing the Potential of Mean Force (PMF) using the COM of BZS molecules as reference, as shown in Figure S37.

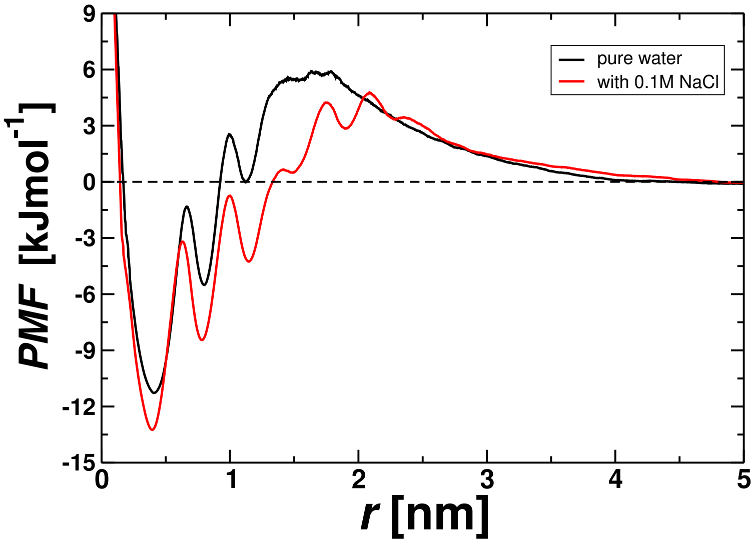

Figure S37: Potential of Mean Force (PMF) between BZS moieties as a function of their COM separation distance in pure water (black) and with 0.1M NaCl (red).

The PMF plot displays multiple alternated minima (contact mimina) separated by an equal number of maxima (desolvation peaks) likely witnessing the propensity of BZS moieties

to form variegated aggregate sizes. Noteworthy, BZS aggregation is a favorable association process (PMF<0) undoubtedly driven by stabilizing pair-wise hydrophobic-hydrophobic contacts, the minimum contact distance being found around 4.2 Å. Interestingly, the contact minimum at 298.15 K in Fig. S37 exhibits a more negative value with salt inclusion, confirming a more favorable energetic interaction in this simulation than in pure water. Further insights on the stability of the tetramer conformation can be obtained by considering a pre-assembled stack of BZS molecules, thus forming a tetramer lattice, and check its stability. Following up the results from previous simulations of free BZS molecules as described in Figure S38, a stable 4mer-stack building block was extracted and used to build three model systems by placing two stacked tetrameric blocks of BZS moieties in three different configurations : one exactly on top of the other on parallel planes forming a sandwich-like arrangement (Figure S38a), one on top of the other on parallel planes but with positions shifted with respect to each other forming a parallel offset-like arrangement (Figure S38b), and the side-to-side configuration wherein the hydrophilic tails of BZS are placed one next to the other (Figure S38c).

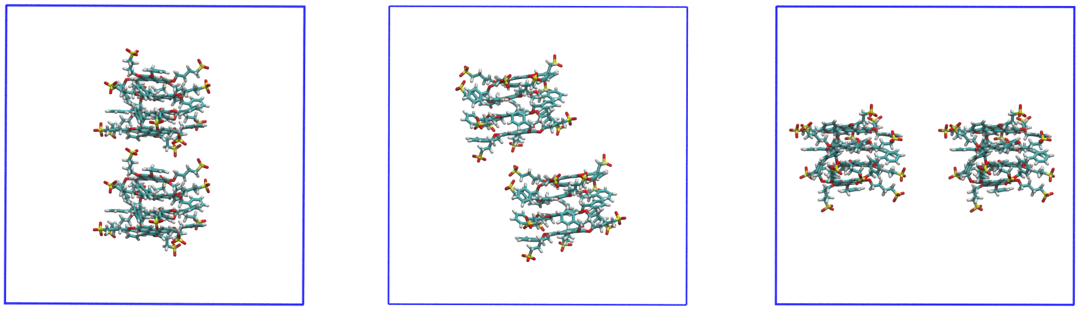

(a) Sandwich-like setup. (b) Parallel off-set display. (c) Side-to-side arrangement.

Figure S38: Overview of the initial starting configurations of BZS stacked assemblies used to further probe the stability of the tetrameric order arrangement corresponding to sandwich-like setup (a), parallel offset arrangement (b), and side-to-side display (c). BZS moieties are shown in licorice representations while solvent and ions are omitted for clarity.

The results from these simulations confirm the relatively stability of the tetrameric BZS assembly since no sign of disruption was observed independently to the setup considered.

Furthermore, no sign of aggregation grow was monitored since no pentamer nor higher order assembly was seen. This could be ascribed to the low BZS concentration considered in these particular model systems and within the simulation timescale achieved here (50 ns).

S3.5 BZS aggregates stabilized by ADL

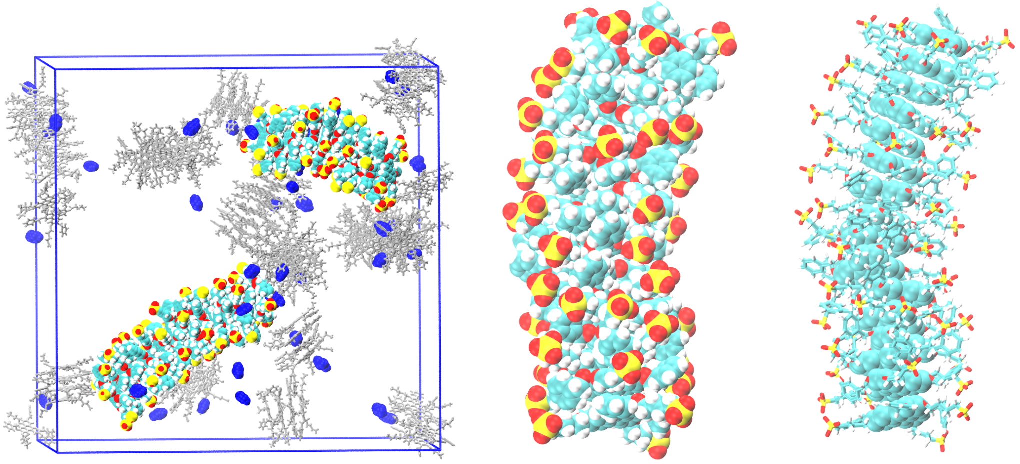

(a) (b)

Figure S39: (a) Overview of the final simulation (1 µs) box with the two largest BZS aggregates highlighted. ADL units are also shown as blue van der Waals spheres while interacting with BZS moieties. No signs of ADL self-assembly are reported. (b) The close-up views of the largest stable cluster *16mer*-BZS identified in the simulation is shown in two different representations with the central hydrophobic patch of BZS highlighted in the second view.

We have also added 41 small binder molecules – adamantane (ADL), and study how this addition interferes with the aggregation process. A representative snapshot obtained after 1 µs of production run is displayed in Figure S39a, where one of the tetramer lattices have been highlighted and all ADL molecules have been put in the limelight as well. The idea underlying the addition of the ADL molecules was the following. Imagine the tetramer lattice to be marginally stable. Then the hydrophobic character of the ADL molecule could compete with the $\pi - \pi$ stacking of different BZS molecules and further destabilize the stacking. The results presented in Figure S39a suggests that this does not seem to be the case. Figure S39b then displays a *16mer* assembly that appears to be stabilized both by hydrophobic interactions, promoting the stacking, and by hydrogen bonding of the external

charge groups with water.

We repeat the same analysis of the order parameters that was carried in Figure 7 of the main text for the single BZS molecules. This is reported in Figure S39. Panel (a) shows the progressive decrease of SASA along the time trajectory, again suggesting aggregation or a deformation of the initial stack, but panel (b) reports the results of the radial distribution function $g(r)$ of the distances between the BZS centers and shows periodic peaks compatible with the layering of the tetralattice hinted by the snapshots in Figure 6a of the main text. Both these findings can also be rationalized by visual inspection of the representative snapshot reported in Figure S39, and this is also confirmed by the absence of a significant drift for the separation distance between adjacent nearest-neighbor oxigen atoms (panel (c)). Finally, the cluster size distribution (panel (d)) shows a strong peak for cluster sizes 4 and 8, and then a decreasing trend for higher clusters until a size of approximately 30. This indicates that the original tetralattice has been deformed and some of the original tetramer units have been rearranged and recombined, in agreement with the decrease in the SASA.

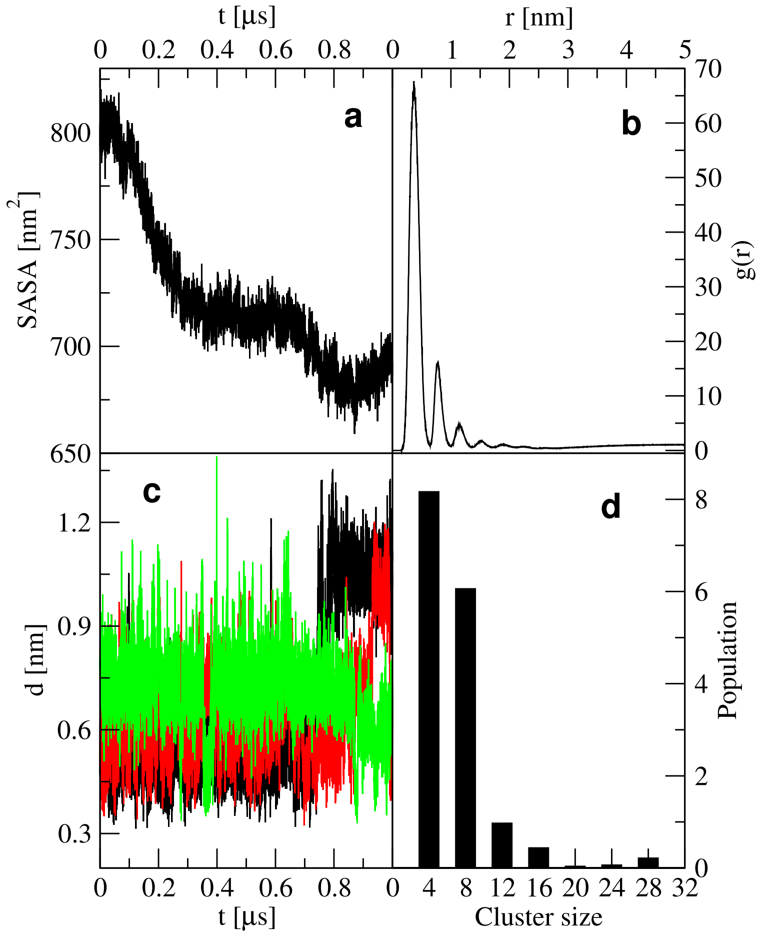

Figure S40: (a) Atomistic simulation results of BZS/ADL model system. The time-based total accessible surface area of BZS entities. (b) The BZS-BZS center of mass pair radial distribution function. (c) The separation distance between adjacent nearest neighbour oxygen atoms of aliphatic oxy-butylsulfonate tails in the 2 centro-mers BZS moieties as a function of time. (d) The cluster size distribution.

References

(S1) Doucet, M.; Adams, M.; Agouzal, N.; Alina, G.; Attala, Z.; Backman, M.; Bakker, J.; Beaucage, P.; Berger, J.; Bourne, R.; Bouwman, W.; Bressler, I.; Butler, P.; Cadwallader-Jones, I.; Campbell, K.; Cho, J.-H.; Cooper-Benun, T.; Cortes Hernandez, R.; Crake-Merani, J. A.; Detiste, A.; Doutch, J.; Dresen, D.; Drosos, G.; Durniak, C.; Farrow, C.; Ferraz Leal, R.; Ford, R.; Forster, L.; Gaudet, J.; Gerina, M.; Gilbert, P.; Gonzalez, M.; Hammond, O.; Hansen, T. B.; Heenan, R.; Henson, S.; Hewins, E.; Honecker, D.; Hicks, A.; Jackson, A.; Jensen, G.; Juhas, P.; Karliczek, J.; Kienzle, P.; King, S.; Kline, S.; Krzywon, J.; Lee, S. B.; Lin, J.; Liu, Y.; Lopes, R.; Lozano, D.; Lytje, K.; Mannicke, D.; Maranville, B.; Markvardsen, A.; Martinez, N.; McKerns, M.; Miller, B.; Mothander, K.; Murphy, R.; Nelson, A.; Nielsen, T.; Oakley, M.; O'Driscoll, L.; Park, H.; Parker, P.; Patrou, M.; Peterson, P.; Potrzebowski, W.; Prescott, S.; Rakitin, M.; Richter, T.; Rooks, J.; Rozyczko, P.; Shan, X.; Snow, T.; Stellhorn, A.; Teixeira, S.; Tumarkin, J.; Washington, A.; Weigandt, K.; Whitley, R.; Wilkins, L.; Wolf, C.; Zhang, A.; Zheng, A. SasView version 6.1.0. 2025; `https://zenodo.org/doi/10.5281/zenodo.15775666`.

(S2) Porod, G. Die Röntgenkleinwinkelstreuung von dichtgepackten kolloiden Systemen: I. Teil. *Kolloid-Zeitschrift* **1951**, *124*, 83–114.

(S3) Claudio, T.; Stein, N.; Stroppa, D. G.; Klobes, B.; Koza, M. M.; Kudejova, P.; Petermann, N.; Wiggers, H.; Schierning, G.; Hermann, R. P. Nanocrystalline silicon: lattice dynamics and enhanced thermoelectric properties. *Phys. Chem. Chem. Phys.* **2014**, *16*, 25701–25709.

(S4) Kratky, O.; Porod, G. Diffuse small-angle scattering of x-rays in colloid systems. *Journal of Colloid Science* **1949**, *4*, 35–70.

(S5) Kline, S. R. Reduction and analysis of SANS and USANS data using IGOR Pro. *Journal of Applied Crystallography* **2006**, *39*, 895–900.

(S6) Higgins, J. S.; BenoÎt, H. C. *Polymers and Neutron Scattering*; Oxford University PressOxford, 1998.

(S7) Guinier, A.; Fournet, G.; Walker, C. B.; Yudowitch, K. L. *Small-angle Scattering of X-rays*; Wiley New York, 1955.

(S8) Michaud-Agrawal, N.; Denning, E. J.; Woolf, T. B.; Beckstein, O. MDAnalysis: A toolkit for the analysis of molecular dynamics simulations. *Journal of Computational Chemistry* **2011**, *32*, 2319–2327.

(S9) Gowers, R.; Linke, M.; Barnoud, J.; Reddy, T.; Melo, M.; Seyler, S.; Domański, J.; Dotson, D.; Buchoux, S.; Kenney, I.; Beckstein, O. MDAnalysis: A Python Package for the Rapid Analysis of Molecular Dynamics Simulations. Proceedings of the 15th Python in Science Conference. 2016.

(S10) Virtanen, P.; Gommers, R.; Oliphant, T. E.; Haberland, M.; Reddy, T.; Cournapeau, D.; Burovski, E.; Peterson, P.; Weckesser, W.; Bright, J.; van der Walt, S. J.; Brett, M.; Wilson, J.; Millman, K. J.; Mayorov, N.; Nelson, A. R. J.; Jones, E.; Kern, R.; Larson, E.; Carey, C. J.; Polat, İ.; Feng, Y.; Moore, E. W.; VanderPlas, J.; Laxalde, D.; Perktold, J.; Cimrman, R.; Henriksen, I.; Quintero, E. A.; Harris, C. R.; Archibald, A. M.; Ribeiro, A. H.; Pedregosa, F.; van Mulbregt, P.; SciPy 1.0 Contributors SciPy 1.0: Fundamental Algorithms for Scientific Computing in Python. *Nature Methods* **2020**, *17*, 261–272.

(S11) Ledum, M.; Sen, S.; Li, X.; Carrer, M.; Feng, Y.; Cascella, M.; Bore, S. L. HylleraasMD: A Domain Decomposition-Based Hybrid Particle-Field Software for Multiscale Simulations of Soft Matter. *Journal of Chemical Theory and Computation* **2023**, *19*, 2939–2952.

(S12) Ledum, M.; Carrer, M.; Sen, S.; Li, X.; Cascella, M.; Bore, S. L. HylleraasMD: Massively parallel hybrid particle-field molecular dynamics in Python. *Journal of Open Source Software* **2023**, *8*, 4149.
